# Prediction, prevention, and management of right ventricular failure after left ventricular assist device implantation: A comprehensive review

**DOI:** 10.3389/fcvm.2022.1040251

**Published:** 2022-11-03

**Authors:** Eduard Rodenas-Alesina, Darshan H. Brahmbhatt, Vivek Rao, Marcus Salvatori, Filio Billia

**Affiliations:** ^1^Mechanical Circulatory Support Program, Peter Munk Cardiac Center, University Health Network, Toronto, ON, Canada; ^2^Ted Roger’s Center for Heart Research, University Health Network, Toronto, ON, Canada; ^3^Department of Cardiology, Vall d’Hebron University Hospital, Barcelona, Spain; ^4^National Heart and Lung Institute, Imperial College London, London, United Kingdom; ^5^Department of Anesthesia, University Health Network, Toronto, ON, Canada

**Keywords:** left ventricular assist device (LVAD), heart failure (HF), right ventricle (RV), right ventricular failure (RVF), right ventricular assist device (RVAD), hemocompatibility adverse events (HRAE)

## Abstract

Left ventricular assist devices (LVADs) are increasingly common across the heart failure population. Right ventricular failure (RVF) is a feared complication that can occur in the early post-operative phase or during the outpatient follow-up. Multiple tools are available to the clinician to carefully estimate the individual risk of developing RVF after LVAD implantation. This review will provide a comprehensive overview of available tools for RVF prognostication, including patient-specific and right ventricle (RV)-specific echocardiographic and hemodynamic parameters, to provide guidance in patient selection during LVAD candidacy. We also offer a multidisciplinary approach to the management of early RVF, including indications and management of right ventricular assist devices in this setting to provide tools that help managing the failing RV.

## Introduction

Left ventricular assist devices (LVADs) are commonly used for patients with end-stage heart failure (HF) as destination therapy (DT), bridge to transplant (BTT) or to orthotopic heart transplant (OHT) candidacy (1). As the right ventricle (RV) is not supported, right ventricular failure (RVF) is a feared complication that occurs in 20–40% patients early after LVAD implantation (2). Right ventricular assist devices (RVADs) are required in approximately 5% of cases (3, 4) with an associated increase in mortality, morbidity, and cost. In patients successfully discharged from hospital, persistent and new-onset late RVF can complicate patients’ clinical course with higher rates of HF admission, mortality, and hemocompatibility-related adverse events (HRAEs). Determining the risk of developing RVF is paramount, as it may be the sole preclusion of LVAD implantation and the only way of planning ahead simultaneous RVAD support. Prediction of RVF is based on clinical variables and direct RV assessment, which integrates echocardiographic and invasive hemodynamic data. Clinical optimization before LVAD implantation, surgical technique for LVAD implantation and concomitant interventions can modify rates of both early and late RVF. Intra-operative management and decision-making about appropriateness, type, and timing of RVAD support is key. The purpose of this review is to provide a practical guideline for patient selection, RVF prognostication and RVF management by incorporating the most recent evidence, that can be used by HF specialists, cardiovascular surgeons, or anesthetists during LVAD candidacy assessment and post-operative period.

## Definition of right ventricular failure

The definition of RVF has been cumbersome with many working groups utilizing their own definition. Since 2014, the Interagency Registry of Mechanical Circulatory Support (INTERMACS) definition of RVF requires documentation of elevated central venous pressure (CVP) and tangible clinical or laboratory manifestations of RVF. INTERMACS grades RVF severity according to the duration of required therapy [mainly nitric oxide (iNO) and inotropes] with 0–7 days of support defined as mild RVF, 7–14 days considered moderate RVF, and >14 days or need for RVAD was defined as severe RVF (5). Subsequent studies have demonstrated that only severe, INTERMACS-defined RVF is associated with worse outcomes (6, 7). Thus, it seems reasonable to consider a clinically relevant episode of RVF as only those episodes that require RVAD or inotropic support for >14 days, acknowledging that prognosis worsens with duration (8, 9).

**FIGURE 1:**
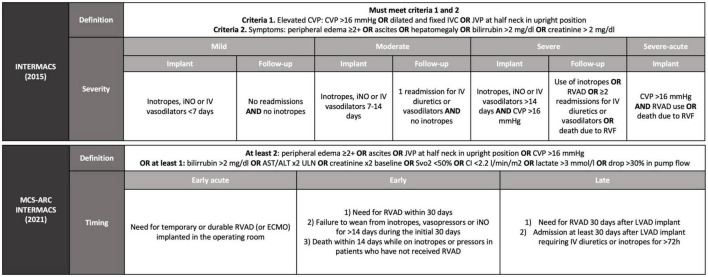
Definition of right ventricular failure after LVAD implantation according to INTERMACS from 2015 and from 2021. INTERMACS, interagency registry for mechanically assisted circulatory support; MCS-ARC, mechanical circulatory support–academic research consortium; CVP, central venous pressure; IVC, inferior vena cava; JVP, jugular venous pressure; iNO, nitric oxide; IV, intravenous; RVAD, right ventricular assist device; RVF, right ventricular failure; ULN, upper limit of normal; Svo2, central venous oxygen saturation; CI, cardiac index; ECMO, extracorporeal membrane oxygenator; LVAD, left ventricular assist device.

The INTERMACS definition, however, fails to capture late RVF, as prolonged inotropic or RVAD support are rarely needed. To account for this discrepancy, an updated definition was released by the Mechanical Circulatory Support–Academic Research Consortium (MCS-ARC) in 2020 (10). The new definition distinguishes between early acute RVF (requiring RVAD support), early RVF in the first 30 days, and late RVF after the first 30 days. For the MCS-ARC definition, RVF is diagnosed in the presence of RVF signs and symptoms in combination with increased diuretic or inotrope requirement for at least 72 h (Figure 1).

## Pre-operative prediction of right ventricular failure

**FIGURE 2:**
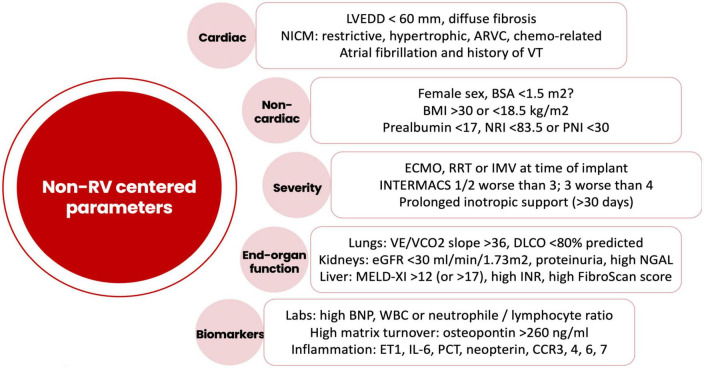
Risk factors for right ventricular failure after LVAD implantation not related to the intrinsic right ventricular function. LVEDD, left ventricular end-diastolic diameter; NICM, non-ischemic cardiomyopathy; ARVC, arrhythmogenic right ventricular cardiomyopathy; VT, ventricular tachycardia; BSA, body surface area; BMI, body mass index; NRI, nutrition risk index; PNI, prognostic nutrition index; ECMO, extracorporeal membrane oxygenation; RRT, renal replacement therapy; IMV, invasive mechanical ventilation; INTERMACS, interagency registry for mechanically assisted circulatory support; VE, minute ventilation; VCO2, carbon dioxide production; DLCO, carbon monoxide diffusion capacity; eGFR, estimated glomerular filtration rate; NGAL, neutrophil gelatinase-associated lipocalin; MELD, model for end-stage liver disease; INR, international normalized ratio; BNP, brain natriuretic peptide; ET1, endothelin 1; IL6, interleukin-6; PCT, procalcitonin; CCR, CC chemokine receptor.

**FIGURE 3:**
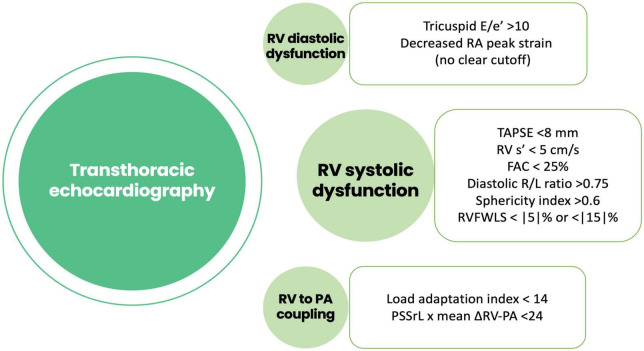
Risk factors for right ventricular failure after LVAD implantation obtained using echocardiography. RA, right atrial; TAPSE, tricuspid annular plane systolic excursión; RV, right ventricle; FAC, fractional área change; RVFWLS, right ventricular free wall longitudinal strain; PSSrL, peak systolic longitudinal strain rate; PA, pulmonary artery.

**FIGURE 4:**
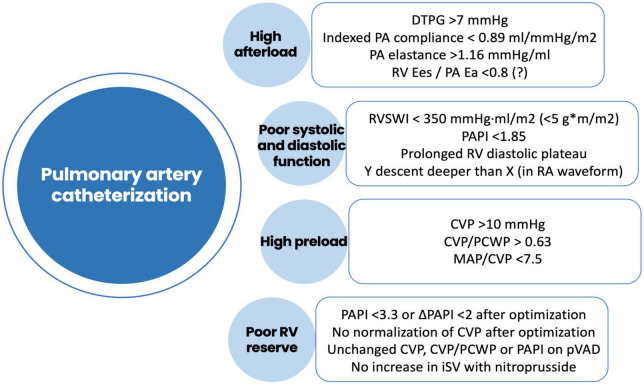
Risk factors for right ventricular failure after LVAD implantation obtained using pulmonary artery catheterization. DTPG, diastolic transpulmonary gradient; PA, pulmonary artery; Ees, end-systolic elastance; Ea, arterial elastance; RVSWI, right ventricular stroke work index; RV, right ventricle; RA, right atrium; CVP, central venous pressure; PCWP, pre-capillary wedge pressure; MAP, mean arterial pressure; PAPI, pulmonary artery pulsatility index; pVAD, percutaneous ventricular assist device; iSV, indexed stroke volume.

Both early and late RVF most likely result from a multiple hit combination of pre-existent RV dysfunction, surgical insult, and RV loading conditions after initiation of LVAD support. Therefore, none of the published predictors has emerged as a standalone gold standard and an integrative approach is required. We advocate for a combination of patient-specific and RV-focused metrics obtained from echocardiography and pulmonary artery (PA) catheterization, summarized in Figures 2–4.

### Clinical risk factors for right ventricular failure

#### Body size and cardiac size

In the MOMENTUM3 trial, the average body surface area (BSA) of patients with LVADs was 2.1 m^2^, a finding consistent across all large-scale, MCS registries (11). Smaller patients have historically been considered high-risk, and industry recommendations do not support the use of LVAD support in patients with a BSA <1.5 or <1.8 m^2^. An INTERMACS analysis of 10,813 patients, however, has shown comparable rates of survival and RVF in patients with BSA <1.5 m^2^ and BSA >1.5 m^2^, a finding replicated in smaller cohorts using bigger devices such as the Heart Mate II (HM2, Abbott, TX, USA) (12–15).

In healthy patients, smaller BSA reflects smaller cardiac size, but this may not hold true in advanced HF, as BSA and left ventricular end-diastolic diameter (LVEDD) correlate poorly (14). Independently of BSA, an LVEDD <60 mm has been repeatedly associated with RVAD requirement and late RVF, worse tricuspid regurgitation (TR) severity, lower LVAD flow and more LVAD alarms in both axial and centrifugal flow pumps (14, 16, 17). This is probably explained by the interventricular septum being more prone to shifting leftwards in smaller hearts at speeds that would otherwise be considered normal for larger cavities, thus distorting RV geometry (18). In the IMACS registry, the higher mortality and RVF observed in women was partially mediated by a smaller LVEDD, but not by BSA (18).

Although surgical implant may be more challenging, smaller patients benefit equally from LVADs. BSA does not impact RVF risk unless the LVEDD is exceedingly small. Individuals with restrictive or hypertrophic cardiomyopathies and smaller LVEDDs may have poor survival and higher risk of RVF. In such patients, a left atrial (LA) configuration of the inflow cannula may be attempted if LA cavity is big enough (19).

#### Obesity and malnourishment

Obesity, defined as body mass index (BMI) >30 kg/m^2^, confers a 40% increased risk of late RVF according to a recent meta-analysis (20). Other published data report up to a 3-fold risk of RVF in obese patients (21, 22). Obesity and congestion are linked through several well-described mechanisms including increased blood volume, inflammation, microvascular dysfunction and myocardial fibrosis, as well as comorbid conditions such as sleep apnea (23). The increased risk of RVF in obese patients is not associated with increased RVAD requirement or mortality.

Conversely, undernutrition at the time of implant is more common in individuals with pre-operative RVF and gut edema, as demonstrated by a higher CVP in malnourished patients (11 vs. 7 mmHg, *p* = 0.002) (24). Poor nutritional status has demonstrated a negative impact on RVF and survival using different metrics (BMI <18.5 kg/m^2^, prealbumin <17 g/L, nutritional risk index <83.5 points, prognostic nutritional index <30 points) (25–27). Thus, as a modifiable risk factor, deficient nutritional status should be identified during LVAD candidacy assessment and actively addressed.

#### Kidney dysfunction

Kidney dysfunction is a strong predictor for mortality and early RVF, and few centers implant LVADs in stage V chronic kidney disease (CKD) [estimated glomerular filtration rate (eGFR), <15 ml/min/1.73 m^2^] (28, 29). In end-stage HF, cardiorenal syndrome can cause or exacerbate CKD via chronically elevated CVP and suboptimal renal perfusion pressure, which could explain its association with RVF during the early post-operative phase (30). Support for this hypothesis comes from the observation that kidney function improves after LVAD in a vast majority of patients, except in those who develop late RVF (28, 29, 31, 32).

In advanced HF, increased neurohormonal activation promotes the reabsorption of blood urea nitrogen (BUN) (33), with BUN levels being more strongly associated with early RVF than creatinine and eGFR (34). Muscle wasting and sarcopenia will also lower serum creatinine for a given GFR, explaining the paradoxical improvement in eGFR often seen in admitted patients. Cystatin C, a renal marker not affected by cachexia, better predicts RVF and RVAD need in the early post-operative period (35). Neutrophil gelatinase-associated lipocalin (NGAL) is an experimental marker that can differentiate intrinsic tubular damage from that attributable to hemodynamic disturbances, and can also predict an increased risk of RVF (36).

Proteinuria identifies patients with established CKD and faster disease progression and should be routinely tested during LVAD candidacy assessment. A positive dipstick or >0.55 mg protein/mg creatinine doubles the risk of renal replacement therapy (RRT), need for RVAD and mortality (37, 38).

#### Liver dysfunction

Liver dysfunction is another form of end-organ dysfunction attributable to RVF. Like eGFR, liver function tests frequently improve and remain within normal range for years after LVAD implantation, reflecting the close link with venous congestion (39, 40). The MELD score is a chronic liver disease severity scoring system that integrates both liver and kidney function, making it an excellent tool to assess RVF-related congestion before LVAD. Multiple studies have shown an association between MELD scores >12 and early RVF, RVAD support and mortality, although no such association exists for late RVF (41–45). In the absence of warfarin treatment, the international normalized ratio (INR) is an excellent marker of synthetic liver function strongly associated with early RVF (46).

A liver biopsy may distinguish functional liver damage vs. established scarring, that may reflect a primary liver disease or sustained right-sided congestion. In patients with periportal fibrosis (stage F1 and F2 on the biopsy), LVAD has been used successfully (47, 48). There is no data about patients with F3 or F4 fibrosis, but FibroScan elastography has identified higher rates of early RVF in patients with increased pre-operative liver stiffness, most of them within the range of severe fibrosis (24.6 KPa vs. 9.5 KPa, >17.6 KPa being the cutoff for cirrhosis) (49).

#### Pre-operative clinical profile

The number of LVAD implants in patients with INTERMACS 1 and 2 is decreasing over the years, as it is associated with greater RVF and mortality (50). When temporary MCS is used in INTERMACS 1 patients to restore hemodynamics, prognosis with regards to RVF remains equally poor, which may be related to only partial recovery of end-organ function or a more challenging assessment of RV dysfunction while on extracorporeal membrane oxygenator (ECMO) support (51). Rates of temporary RVAD utilization after ECMO is approximately 20% in multiple registries, and some authors even advocate for planned temporary RVAD support in all INTERMACS 1 patients to avoid the second surgery required for staged implantation (50, 52). In patients who cannot be weaned off ECMO, LVAD may be non-inferior to OHT, but assessment of RV function is important in deciding whether to proceed to one of these two high-risk alternatives (52). In patients who are receiving temporary MCS, assessment of RV function and reserve is more complicated due to the decrease in RV preload [in ECMO or biventricular assist device (BiVAD)] or afterload [in isolated left ventricle (LV) support], and a pulmonary artery catheter (PAC)-guided management could help in patient selection and even improve survival, as shown in a recent meta-analysis of observational data (53).

Interagency registry of mechanical circulatory support three patients, and especially those with ongoing inotropic support for >30 days, also tend to develop more RVF after the surgery than INTERMACS 4 or higher. This may be a marker of RVF pre-operatively and could identify patients with little margin for improvement solely using pharmacological adjustments if early RVF occurs (54, 55). Lastly, LVAD implantation should be considered cautiously in patients with ongoing invasive mechanical ventilation or RRT, as these have been consistently associated with early RVF (46, 56, 57).

#### Biomarkers and the role of inflammation

There is a growing interest in biomarkers as predictors of RVF. Elevated BNP reflects stretch of myocardial fibers and has been correlated with higher RVF rates (58). Increased osteopontin (>260 ng/ml) is a marker for greater extracellular matrix turnover and identifies patients with an ongoing profibrotic process that may potentially involve the RV, with increased risk of RVF (59). Patients with RVF also display persistently high osteopontin levels after surgery and have less reverse remodeling (60). Direct measurement of this fibrotic process in myocardial tissue from the apical core removed at the time of LVAD implant has showed that increased collagen type 1 mRNA expression is associated with RVF and need for RVAD (61). Non-invasive measures of diffuse fibrosis such as cardiac MRI with T1 mapping have not been evaluated for this purpose.

Inflammation may be a relevant mediator of RVF in early phases. Readily available inflammatory markers such as white cell count, neutrophil to lymphocyte ratio, or C reactive protein correlate with rates of RVF (62). A more in-depth analysis showed that patients with RVF displayed a pre-operative downregulation of chemokine receptors CCR3, 4, 6, 7, and 8 that was even more profound in patients who required RVAD, which suggests a dose-response relationship that supports a causative role (63). Other inflammatory biomarkers such as procalcitonin, neopterin, or endothelin 1 have also been linked to RVF (64). As worse INTERMACS profiles have higher inflammatory biomarkers [such as interleukin-6 (IL-6)], the association between INTERMACS profile and RVF could be partially mediated by inflammation (65, 66). The therapeutic benefit of treating inflammation before LVAD implant in high-risk cohorts has not been yet explored.

#### Respiratory disorders

Severe respiratory disease leads to increased pulmonary vascular resistance (PVR), a major contributor to RVF. Surprisingly, current evidence suggests that chronic obstructive pulmonary disease is not associated with overt RVF and does not impact mortality after LVAD, although quality of life and functional capacity remain compromised (67). Central sleep apnea frequently resolves with the increase in cardiac output (CO) provided by the LVAD, but obstructive or mixed episodes may persist and even cause nocturnal drops in LVAD flows (68), presumably related to transient hypoxia-mediated increases in PVR (69). A pre-operative carbon monoxide diffusion capacity <80% identified patients whose PVR will not decrease after surgery and who are at higher risk of recurrent HF admissions (70). Finally, patients with elevated ventilatory efficiency (VE)/carbon dioxide output (VCO2) slope on a cardiopulmonary exercise test (a marker of inefficient ventilation that may indicate disproportionate increase in PA pressures during effort) had a close association with RVF, higher CVP and lower pulmonary artery pulsatility index (PAPI), whereas peak oxygen consumption (VO2) had no association (71).

#### Other clinical risk factors for right ventricular dysfunction

In myocardial diseases with primary involvement of the RV, such as arrhythmogenic right ventricular cardiomyopathy with severe RV dysfunction and dilatation, LVAD support will provide minimal benefit. Other cardiomyopathies impact the RV in a less apparent way, explaining the higher rates of RVF of non-ischemic cardiomyopathy (NICM) compared to their ischemic counterparts. This is most evident for NICM related to chemotherapy, which damages both ventricles, and is associated with higher rates of RVAD utilization (72, 73). Atrial fibrillation is marginally associated with RVF, presumably due to the detrimental effect of losing atrial contraction on CO (74). Subjective assessment of lower limb hemosiderosis has been used by some as a surrogate for long-standing right-sided congestion (75).

### Right ventricular function assessment

In HF, RV dysfunction is usually related to chronically elevated left-sided filling pressures and secondary pulmonary hypertension, which constitutes a hallmark of advanced HF and is related to the remodeling of the pulmonary vasculature (76). The adaptative response of the RV is an increase in contractility to match the increased afterload, and RV hypertrophy or dilatation may occur. If RV afterload remains high, RV contractility may not be able to overcome the imposed resistance, leading to an uncoupling of RV and PA pressures. When RV-PA uncoupling occurs without correction of the RV afterload, RVF may result. As RV contractility worsens, PA pressures drop as the RV fails to generate an appropriate stroke volume. In severe RVF, even the correction of afterload will not improve RV contractility, and RVAD support may be required. This bimodal relationship between PA pressures, RV-PA uncoupling and RV contractility and dilatation explains most of the echocardiographic and hemodynamic predictors that have been studied. In all-comers with HF, higher PA pressures are usually associated with worse outcomes (77). However, in patients undergoing LVAD candidacy assessment, higher PA pressures confer a better prognosis, as low PA pressures are usually associated with a drop in RV contractility (78).

#### Echocardiographic risk factors for right ventricular dysfunction

##### Right ventricular systolic function

Most echocardiographic predictors of RVF are based on RV systolic dysfunction, although all of them are afterload dependent and portend higher rates of RVF in the setting of a low afterload. Despite the existence of different cutoffs for RVF risk, all predictors should be approached as a continuum of risk rather than a dichotomous value. Tricuspid annular plane systolic excursion (TAPSE) less than 8 mm has been consistently associated with RVF risk, increasing the predictive capacity of different risk scores (79–81). The suggested cutoff to predict RVF for RV’s tissue Doppler s′ was <5–8 cm/s (82, 83). RV fractional area change (FAC) accounts for radial contraction in addition to longitudinal contraction, and is associated with RVF <25–30% (79, 84). RV dilatation can be assessed with the ratio between RV and LV end-diastolic diameters in a 4-chamber view, or right-to-left ratio. Another useful tool is the ratio between the end-diastolic RV mid-ventricular and longitudinal diameters, or the sphericity index. The risk of RVF increases when the right-to-left ratio is >0.72–0.75 (85, 86), or when the sphericity index is >0.6 (82, 87, 88). The contribution of septal fibers to RV contractility is severely reduced after LVAD implant, and the assessment of RV free wall contractility with deformation techniques predicts RVF with accuracy similar to conventional RV systolic assessment. However, the cutoffs for RV free wall longitudinal strain for predicting RVF vary widely across studies, ranging from <5 to <15%, which precludes practical implementation (46, 79, 89–93). The capacity of simultaneous multiplanar echocardiography and 3-dimensional imaging to evaluate the complex RV anatomy may improve discrimination (88, 94–96).

##### Right ventricular to pulmonary artery coupling

Indices of RV systolic function can be misleading, as they can be affected by excessive afterload independent of intrinsic RV dysfunction. Measures of RV-PA coupling such as TAPSE/systolic PA pressure that have been shown to be predictive of RVF in all-comers with HF do not reliably predict RVF in the LVAD population (97). This is not surprising, as a low TAPSE/systolic PA pressure identifies patients with mild RV-PA uncoupling (initial drop in systolic function related to elevated RV afterload) but cannot capture patients with more advanced stages of RV-PA uncoupling (drop in systolic PA pressure due to a decrease in RV stroke volume), who are precisely the patients at higher risk of post-operative RVF. The product of peak systolic longitudinal strain rate × mean RV-PA gradient <24 or a load adaptation index [(mean RV-PA velocity time-integral × longitudinal RV diameter)/RV end diastolic area] <14 identify patients with a failing RV unable to generate appropriate PA pressures, and both indexes have a very high accuracy in predicting RVF in candidates for LVAD implantation (82). Several other metrics of late-stage RV-PA uncoupling have been tested in HF population, but their usefulness remain to be proven in LVAD recipients (98).

##### Right ventricular diastolic dysfunction

Diastolic RV dysfunction is frequently overlooked as a predictor of RVF following LVAD implant. A restrictive RV filling pattern and/or high filling pressures can be assessed with the tricuspid E/e′ ratio, which predicts RVF when >10 in the 72 h preceding LVAD surgery (83). Chronically elevated RV end diastolic pressure is better captured by peak longitudinal right atrial (RA) strain. The only study analyzing RA strain showed striking differences between groups (average peak RA strain 11% for those with RVF vs. 33% for those without, *p* < 0.01), with excellent discrimination [area under the curve (AUC) = 0.913] to predict the need for RVAD (99).

#### Invasive assessment of right ventricle to predict right ventricular failure

##### Right ventricular afterload

Invasive hemodynamic measurement using a PA catheter is the gold standard for assessment of RV afterload. The transpulmonary gradient (TPG) [mean PA pressure–pulmonary capillary wedge pressure (PCWP)] or the transpulmonary diastolic gradient (TPDG) (diastolic PA pressure–PCWP) are simple measures that can identify a fixed component of RV afterload likely to persist after LVAD implant. They are of limited use because they do not account for pulmonary flow. A TPDG >7 mmHg predicted RVF after LVAD implantation in a single study (100).

Right ventricle afterload can be divided into a resistive component and a pulsatile load. The resistive component of RV afterload is captured by PVR [(mean PA pressure–PCWP)/cardiac output (CO)], but PVR has not been consistently associated with worse RV function after LVAD. Compliance, calculated as [stroke volume/(systolic PA pressure–diastolic PA pressure)], measures pulsatile RV load, and may be a better marker of RV afterload in HF patients that is highly sensitive to changes in PCWP (101). When indexed by BSA, compliance <0.89 ml/mmHg/m^2^ predicts RVF after LVAD (7, 102). Finally, PA elastance (Ea) incorporates both pulsatile and resistive components, and may be a better measure of global RV afterload (103). PA elastance is calculated as systolic PA pressure/stroke volume, and outperforms PA compliance for predicting RVF after LVAD with a suggested cutoff of >1.16 mmHg/ml (7).

##### Right ventricular systolic and diastolic function

Assessing intrinsic RV systolic performance independent of other variables remains challenging. The parameter most often used for this purpose is right ventricular stroke work index (RVSWI), a flow-dependent estimate of RV contractile function. RVSWI is calculated as [mean pulmonary artery pressure (mPAP)–CVP] × stroke volume index (SVI) × 0.0136. An RVSWI >5 g × m/m^2^ (equivalent to 350 mmHg⋅m*^l^*/m^2^) is predictive of RVF after LVAD implant (46, 104). Other proposed load-independent metrics include RV dp/dt and direct estimation of end-systolic elastance (Ees) using pressure-volume loops, but there is only preliminary data using these parameters and no proposed cutoffs (105, 106).

Right ventricle diastolic dysfunction cannot be directly measured using standard monitors, which limits clinical utility. However, the RA pressure waveform provides a readily available surrogate of RV distensibility. In the absence of TR, a Y descent deeper than the X descent reflects impaired RV diastolic relaxation. Qualitative RA waveform assessment has excellent interobserver reliability, with impaired RV relaxation seen in 20% of LVAD candidates. A non-distensible RV is a strong predictor of early RVF, and in a small cohort was observed in all patients requiring RVAD support and in those with late RVF (107).

##### Right ventricular preload

Systolic and diastolic dysfunction will eventually lead to inefficient volume management with a resultant increase in preload and CVP. Elevated pre-operative CVP (>10–14 mmHg depending on the study) has been consistently associated with RVF after LVAD (46, 104). Furthermore, CVP increases with vasoplegia, often seen in combination with RVF (108). The CVP/PCWP ratio >0.63 distinguishes between volume overload vs. disproportionate RVF as the cause for elevated CVP. A mean arterial pressure (MAP)/CVP ratio <7.5 may also suggest high RV preload and higher risk of RVF (109).

##### Integrative measures of right ventricular assessment

As with echocardiographic evaluation, an integrative approach incorporating multiple invasive hemodynamic parameters is likely to improve RVF risk prediction. Pulmonary artery pulsatility index (PAPi) (systolic PA–diastolic PA)/CVP is PAC-derived measurement that reflects both preload and afterload with excellent predictive capacity for RVF at values <1.85–2 (91, 104, 110, 111). PAPi <1.85 is associated with higher rates of RVF, need for RVAD and mortality. Moreover, PAPi may reflect an abnormal myocardial substrate, as it has also been linked to RV sarcomere contractile dysfunction (112). Models that integrate indices of RV distensibility, PA compliance and PA elastance more accurately predict post-operative RVF from diastolic dysfunction or afterload (7, 102, 107). Likewise, patients with both RVSWI <5 g × m/m^2^ (low contractility) and elevated PVR (elevated resistance) are at significantly higher risk of RVF and RVAD (113). Other integrative measures of RV-PA coupling such as Ees/Ea ratio (normal values >0.8) have shown excellent prognostic value in advanced HF population (114) but have not been studied to predict RVF in LVAD patients.

##### Assessment of right ventricular reserve

Right ventricle function, preload and afterload are dynamic variables that may fluctuate pre- and post-LVAD implantation, and these static metrics may be unable to fully account for RV reserve. In a moderately large cohort, baseline PAPi predicted RVF, but an optimal PAPi <3.3 after hemodynamic optimization or an increase in PAPi (ΔPAPi) <2 had a much stronger predictive value for RVF (115). Similarly, PAPi measurement while on inotropes better predicts RVF than when measured off inotropic support (111). The same findings were observed for CVP, as patients with normalized CVP after hemodynamic optimization had the same outcomes as those with low CVP at admission (116). The strong predictive value of pre-operative hemodynamics raises the question of whether pre-emptive percutaneous RVAD support for hemodynamic tailoring and RV unloading could improve outcomes in patients at very high-risk for RVF (117). A vasodilator challenge with sodium nitroprusside (SNP) is also helpful in predicting RVF, so that PAPi after a SNP administration was the strongest predictor of RVF in a small prospective study (118). In the same line, a multicentric collaboration also demonstrated that a blunted increase <22 m*^l^*/m^2^ in indexed stroke volume after SNP challenge was consistently associated with higher rates of RVF (119).

In patients at high risk of RVF, RV reserve can be assessed using a temporary, percutaneous LVAD. Within 48–72 h of insertion of an Impella CP (Abiomed, Danvers, MA, USA), all parameters of RV afterload improve (reduction in PCWP, PVR and Ea), as does CVP and CVP/PCWP (120). The ratios Ea/CVP and Ea/(CVP/PCWP) remain unchanged as they reflect the RV capacity to cope with a given afterload. These metrics may therefore have higher value in the static assessment of RV function (120). These results were replicated in patients bridged to LVAD with axillary Impella devices (121). Conversely, patients with little or no improvement in CVP, PAPI and CVP/PCWP ratio after Impella support developed RVF (121).

#### Scores to predict right ventricular failure

Currently, there are over 20 published RVF risk scores that integrate clinical, echocardiographic, and hemodynamic variables, but few have undergone external validation. The most commonly used scores are the HM2 Risk Score, CRITT, EUROMACS, Michigan and ALMA scores (56, 57, 122–124). Most of these scores were developed using single center registries and axial flow pumps, display modest discrimination capacity (AUC 0.68–0.74), and performed poorly when externally validated (AUC 0.53–0.65) with inaccurate calibration (2). Overall, the currently available risk scores are too unreliable to aid in decision-making but remain a useful tool when comparing average risk of RVF among registries.

A meta-analysis studying predictors of RVF showed that the most robust variables were high CVP, low RVSWI, low MAP, high INR, white blood count and NT-proBNP, qualitative assessment of RV function, higher RV/LV ratio and RVFWLS, as well as pre-operative mechanical ventilation or RRT (46). A Bayesian analysis of the INTERMACS score was also developed for acute, early and late RVF and identified >30 variables with different weights for each scenario, with excellent predictive value (AUC 0.83–0.9). Systolic PA pressures and inflammatory markers had more weight in predicting early RVF whereas PVR and MELD score were more relevant for late RVF (125).

## Surgical considerations to minimize risk of right ventricular failure

### Avoidance of pericardiotomy and cardiopulmonary bypass

The conventional surgical approach to LVAD implantation involves complete midline sternotomy, open pericardium, aorto-bicaval cannulation and cardiopulmonary bypass (CPB). Implantation is most often performed on CPB with a beating heart, as avoidance of cardioplegia and cardiac arrest may lessen post-CBP stunning of the RV.

After surgical LVAD placement, RV adaptation to afterload drastically diminishes, with higher CVP and CVP/PCWP ratios for a given PA elastance. This decompensation is not seen after percutaneous LVAD insertion, highlighting the deleterious effects of surgery (120, 126). CPB, cardioplegia and extensive pericardiotomy can worsen RV dysfunction and dilation and distort the RV geometry, worsening RV-PA uncoupling. In serial echocardiographic assessments during cardiac surgery, there was a 50% drop in RV contractility and increased LV compliance at the time of pericardiotomy, prior to the initiation of CPB (127, 128). RV function further deteriorated with the septal dyskinesis commonly seen upon discontinuation of CPB (129). Cytotoxin liberation during CPB may also negatively impact RV function (62, 63, 65, 66, 130).

The feasibility of a minimally invasive approach was demonstrated in the LATERAL trial, a non-randomized, single arm, prospective trial in which 144 LVAD candidates underwent on-pump HVAD placement through a lateral thoracotomy (LT) and mini-sternotomy, with only 1 patient requiring RVAD support (131). Notably, pre-existent severe RVF was an exclusion criterion for the trial. Although the HeartWare HVAD device (Medtronic, Minneapolis, MN, USA) is no longer available, the feasibility of LT approach avoiding pericardial opening has been demonstrated for Heart Mate III (HM3, Abbott, Chicago, IL, USA) (132, 133). LT was approved by the food and drug administration (FDA) for HM3 in 2020 and is endorsed by industry after the successful LT implant in 44 patients from the ELEVATE registry and in 13 patients from the LAT feasibility study. Off-pump LT implantation is also feasible, but experience is by far less common (134, 135).

The benefits of LT and pericardial preservation on RVF by preserving RV geometry and avoiding distension are still under debate. Whereas a recent INTERMACS analysis using propensity matching did not identify any benefit regarding RVF incidence (136), two contemporary meta-analyses comparing LT to median sternotomy showed a reduction of RVF, RVAD and blood product utilization by more than 50% with almost no heterogeneity in results (137, 138).

### Bleeding and coagulopathy

Intra-operative bleeding and transfusion are common during LVAD implantation and negatively affect RV performance through cytokine release, increased PVR and volume overload. It is estimated that with transfusion of each unit of blood products (red blood cells, platelets or frozen plasma), there is a 10% increase in RVF risk, so that judicious use of blood products is recommended (139, 140).

The use of rotational thromboelastometry (ROTEM) reduces blood transfusions and bleeding in patients undergoing cardiac surgery, and should be considered standard of care during LVAD surgery (141). Baseline ROTEM before surgery can identify primary bleeding disorders or platelet dysfunction that can be addressed before coagulopathy ensues (142). Upon discontinuation of CPB, ROTEM allows for rapid, point-of-care analysis of hemostasis and coagulopathy, enabling targeted transfusion strategies that avoid the indiscriminate administration of blood products.

The main risk factors for intra-operative transfusion are previous cardiac surgery and low pre-operative hemoglobin (139). Pre-operative hemoglobin optimization strategies have been shown to reduce blood product utilization during surgery (143). In patients with unresolved coagulopathy, consideration may be given to packing the mediastinum and leaving the sternum open for delayed closure. Delayed sternal closure allows to control the coagulopathy in the intensive care unit (ICU), minimizing bleeding and avoiding tamponade and the associated excessive pressure on the RV from edematous tissues, thus potentially preventing RVF. This approach, however, has not demonstrated any benefit in the rates of RVF in observational cohorts, prevents early extubation and increases risk of infection (144–146). The peritoneum can be damaged while tunneling the LVAD driveline, allowing peritoneal fluid into the thoracic cavity. This should be avoided, as peritoneal fluid contains tissue plasminogen activator that can worsen coagulopathy (147).

### Tricuspid regurgitation

**FIGURE 5:**
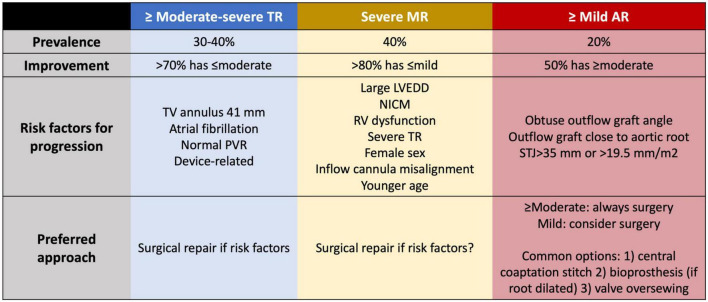
Prevalence, risk factors for progression and management for valvular regurgitation at the time of LVAD implant to minimize the risk of right ventricular dysfunction. TR, tricuspid regurgitation; MR, mitral regurgitation; AR, aortic regurgitation; TV, tricuspid valve; PVR, pulmonary vascular resistance; LVEDD, left ventricular end-diastolic diameter; NICM, non-ischemic cardiomyopathy; STJ, sinotubular junction.

Moderate to severe TR is found in 30–40% of LVAD candidates (Figure 5) (148, 149). If left unrepaired, >70% of cases will regress to less than moderate during follow-up, likely related to the relief of RV afterload, whereas 10% of LVAD patients will develop *de novo* significant TR (150, 151). As expected, severe TR after LVAD is independently associated with RVF and mortality (150). Routine tricuspid valve (TV) repair for severe TR at the time of implantation does not improve long term outcomes, with 30% of TV repairs failing within 1 year of implant (152, 153). Instead, TV repair has been linked with increased risk of arrhythmia, stroke, bleeding, need for reoperation, higher need for RRT and longer stay on mechanical ventilation and in ICU, with no improvement in quality of life thereafter (148, 154). However, the techniques of repair vary widely and have not been standardized. Failure to demonstrate a benefit of TV repair may be associated with a failure of the repair itself or with failure of the selected strategy.

Predictors of persistent TR or *de novo* TR are tricuspid annulus dilatation >41 mm (153, 155, 156), atrial fibrillation (157–159) and normal PVR (159). Despite the risks, repair in these cases is justified to minimize the likelihood of late RVF. In patients with intracardiac devices and suspected device-related severe TR, a surgical exploration seems reasonable if the echocardiography shows a lead adherent or impinging the TV leaflets, as the chance of TR improving if left uncorrected is much lower (160). However, an isolated annuloplasty band for severe TR related to a pacing lead will predictably fail.

### Mitral regurgitation

Significant mitral regurgitation (MR) is observed in 40% of patients at the time of LVAD implantation (Figure 5), but >80% experience improvement to mild or less with adequate LV unloading (161). Persistent MR after LVAD is associated with higher mortality, HF admissions, RVF, severe TR and increased PVR with elevated CVP and PA pressures, and also with worse kidney function (162–166). Patients who received a previous transcatheter edge-to-edge repair can safely undergo LVAD implantation without the need to remove the device if the mean transvalvular gradient remains below 6 mmHg. Compared with patients without transcatheter repair and severe MR at the time of LVAD implantation, previous repair was associated with greater reduction in TPG (167).

Mitral valve repair at the time of LVAD implant is not universally recommended given the high percentage of patients who improve without intervention. Review of the INTERMACS registry shows that mitral valve repair is associated with less MR during follow up, fewer HF admissions, and improved functional capacity and quality of life (161, 164, 168, 169). Risk factors for significant MR after LVAD include severe MR before LVAD, NICM with large LVEDD, RV dysfunction, significant TR, atrial fibrillation, female sex, younger patients, and HM2 support (vs. HM3) (164, 170).

Left ventricular assist device implantations are guided by transesophageal echocardiography (TEE) so that the inflow cannula is oriented toward the mitral valve to favor proper unloading. Using a conventional chest X ray, an inflow cannula with coronal angle >65–75° for HM2 and HVAD and <28° for HM3 is associated with worse unloading, greater MR severity, lower PAPI, less decrease in PCWP per each 100 rpm LVAD speed increase and more HF admissions due to late RVF (171–174). The degree of residual MR following LV unloading can be immediately assessed during surgery, and patients with tenuous RV function and risk factors for MR persistence may benefit from simultaneous mitral valve repair. If LVAD is being used as a bridge to recovery, mitral valve repair also helps to achieve better hemodynamics.

### Aortic regurgitation

Aortic regurgitation (AR) causes a closed-loop circulation between the aorta and LV, and AR following LVAD is clearly associated with higher mortality, more HF admissions, RVF, lower PAPI and higher CVP, all of which are related to increased PCWP (175–177). Any more than mild AR needs to be surgically addressed at the time of LVAD implantation, a situation encountered in 20% of patients (Figure 5) (176, 178). More than half of patients with unrepaired mild AR progress to moderate or severe AR during their time on support (176, 179, 180). Management options include aortic valve repair, bioprosthetic aortic valve replacement, patch closure of the aortic root, complete aortic valve closure, and central aortic valve closure/Park’s stitch (181). Aortic valve closure is associated with higher 2-year mortality than repair or replacement and increases the risk of sudden death in case of device malfunction. Instead of complete closure, repair with a central coaptation stitch ensures partial aortic valve opening. 20% of repairs display recurrent AR vs. 9% of replacements (182). Risk factors for progressive AR following LVAD include a dilated aortic root or sinotubular junction (>19.5 mm/m^2^ or 35 mm), obtuse outflow graft configuration directed toward the aortic valve, and implantation closer to the aortic root (180, 183–187).

Patients who develop AR have higher BNP, reduced cardiac index, higher LVEDD and reduced PCWP decrease at higher LVAD speeds (175, 176), which subsequently increases RV afterload and leads to worse functional class, more frequent HF admissions and worse survival (176, 177, 179). Main predictors for AR are female sex, duration of support and aortic valve closure, which causes commissural fusion of the leaflets (176, 188). AR severity should be routinely monitored during follow-up of LVAD patients using the novel diagnostic criteria that use pulsed wave in the outflow graft for their better correlation with clinical endpoints (189, 190). Aortic valve intervention should be considered when *de novo* AR is at least moderate, there are congestive HF symptoms and hemodynamic ramps suggest poor unloading at increasing speeds. Although a high-risk procedure, transcatheter aortic valve implantation (TAVI) or percutaneous closure using an Amplatzer device (Abbott, Chicago, IL, USA) (191–193) can be performed (194–197), but acute resolution of AR may cause LV collapse with marked leftwards shift and acute RVF in around 15% of reported TAVI procedures (194).

### Coronary artery disease

Damage or occlusion of the right coronary circulation may lead to catastrophic RVF (198, 199). In patients with ischemic cardiomyopathy, and especially in those with previous bypass, assessment of coronary anatomy is necessary to determine RV blood supply and risk of re-entry. In patients with proximal right coronary artery (RCA) occlusion, the RV may rely on collateral branches susceptible to injury during coring for LVAD insertion or sternotomy. Likewise, septal perforators must be preserved to preserve the septal contribution to RV contraction (200, 201). Simultaneous coronary artery graft bypass, however, may be associated with more risks than benefits and there is a very low rate of coronary events in the long-term (200, 202), so that protection of RV circulation should be assessed case-by-case. Severe proximal RCA stenoses limiting coronary perfusion to the RV should likely be addressed at the time of LVAD implant.

## Early and acute right ventricular failure

### Mechanisms of early and acute right ventricular dysfunction

**FIGURE 6:**
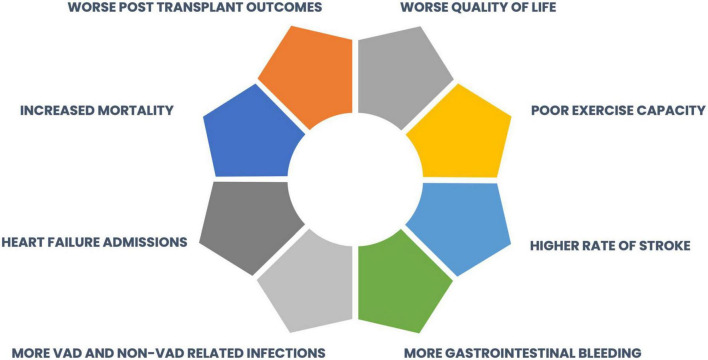
Adverse events associated with right ventricular failure after LVAD implantation.

Acute severe RVF with RVAD requirement is the most catastrophic form of RVF and occurs in approximately 5% of LVAD implants (3, 4). Early RVF and RVAD are associated with multiple complications including prolonged ICU length of stay, acute kidney injury (AKI) and RRT, bleeding and bowel ischemia, stroke, and in-hospital mortality (Figure 6) (203–209). While on CPB, the RV is completely unloaded, and weaning from CPB should be gradual and coordinated with progressive increase in LVAD speed to avoid RV distension. RV geometry and pressure-volume relationships are altered after pericardiotomy, and transient RV dysfunction is common post-CPB. The septal contribution to RV contraction is decreased as the septum is suctioned leftwards, and TV geometry may be distorted, causing a greater regurgitant jet with increased RV dilation, wall tension with higher VO2, and demand ischemia (210). The RV becomes afterload sensitive after LVAD (126), and high pulmonary pressures may compromise function. Early RVF is characterized by a drop in peak RV systolic pressure and RV dp/dt (211). A commonly theorized cause for RV deterioration following LVAD is an increase in RV preload with “flooding” caused by the increased CO. Very limited evidence exists to support this theory, which contradicts the principles of a closed-loop circulation, where the RV output must necessarily match LVAD output.

### Intra-operative assessment of right ventricular performance

After chest closure, invasive measurements of CVP, diastolic PAP and PAPi remain accurate for predicting RVF (212, 213). Although no specific cut-offs have been suggested for clinical use, the relevant values are likely lower than those used in pre-operative RV assessment (212, 213). Patients who develop RVF show a significant intra-operative drop in RVSWI and blunted increase in cardiac index (214). If there is residual pulsatility, pulsus alternans could also be an early sign of RVF after initiating LVAD support (215).

Transesophageal echocardiography can assist in monitoring leftwards septal shift at progressively higher speeds. Intraoperatively, the echocardiographic assessment of RV function described in the pre-operative setting has poor predictive value for acute RVF, with the exception of FAC (213, 216, 217). In the immediate post-operative period, TEE guidance can identify septal misalignment and trigger more speed adjustments than conventional monitoring with a PA catheter, with the most common indicator being a rightwards septum suggesting insufficient unloading (218).

### Decreasing afterload

#### General measures

**FIGURE 7:**
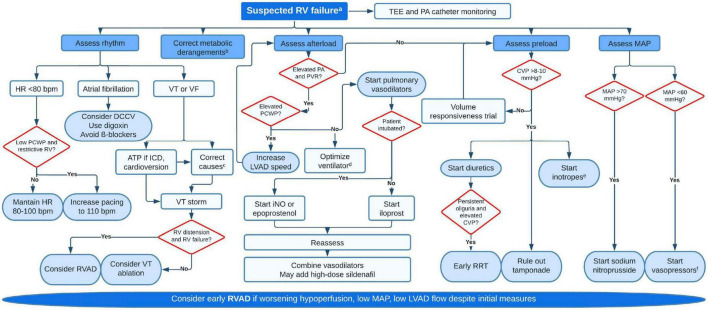
Proposed algorithm for management of early right ventricular failure. ^a^Signs of hypoperfusion (lactate >2 mmol/L, MAP <60 mmHg, drop in pump flows, mottled skin, oliguria) supported by hemodynamic (CVP >15, PAPI <1, RV stroke work index <300, CI <2 ml/min/m^2^, mixed venous oxygen saturation <50%), echocardiographic (dilated RV, leftwards septal bulge, severe TR, fixed and distended IVC) or laboratory data (rise in creatinine, blood urea nitrogen or liver enzymes). ^b^Correct hypoxemia (if significant, rule out intracardiac shunts), hypercarbia and acidosis. ^c^Common causes for VT are metabolic derangements^b^, ischemia (supply demand but also direct damage during surgery), pacing-related issues, scar-mediated or suction events. ^d^Aim for low positive end-expiratory pressure (just enough to minimize atelectasis), low mean airway pressure and avoid air entrapment. Plan for early extubation if possible. ^e^Milrinone (0.125–0.75 μg/kg/min) is usually preferred to dobutamine or epinephrine given their deleterious on PVR. Low dose dobutamine (2–5 μg/kg/min) may not affect PVR. Epinephrine can be used if patient is hypotensive. ^f^Norepinephrine (0.01–0.5 μg/kg/min) is the most used pressor, but vasopressin (0.01–0.06 U/min) should be added at low dose as it has less vasoconstrictive effect on the pulmonary vasculature. RV, right ventricular; TEE, transesophageal echocardiography; PA, pulmonary artery; HR, heart rate; PCWP, pulmonary capillary wedge pressure; DCCV, direct current cardioversion; VT, ventricular tachycardia; VF, ventricular fibrillation; ATP, antitachycardia pacing; ICD, internal cardiac defibrillator; RVAD, right ventricular assist device; PVR, pulmonary vascular resistance; LVAD, left ventricular assist device; iNO, nitric oxide; CVP, central venous pressure; RRT, renal replacement therapy; MAP, mean arterial pressure.

Following separation from CPB, the general principles of RV optimization apply, with reduction of RV afterload being paramount (219). Thorough de-airing is essential as air emboli have a predilection for the RCA due to its anatomic position. Maintaining adequate RV perfusion pressure can be challenging in the context of refractory vasoplegia or use of inodilators (milrinone, dobutamine). Hypercarbia, hypoxemia, acidosis, hypothermia, pain and light anesthesia can all promote pulmonary vasoconstriction and increase PVR, with a detrimental effect on LV filling and LVAD flows (220). Return of CPB volume and subsequent transfusion should be performed slowly and with TEE guidance to prevent RV overload. Protamine reactions can cause significant increases in PVR through thromboxane release (221). For patients with tenuous RV function, intra-aortic administration of protamine bypasses the pulmonary circulation and minimizes its hemodynamic effects (222). Unexplained hypoxemia after LVAD raises the possibility of an unrecognized patent foramen ovale with right to left shunt. Low LVAD speeds may cause insufficient LV unloading and should be adjusted under echocardiographic guidance to reduce RV afterload. Notably, many factors may impact RV resistive load during surgery and general anesthesia, and diastolic PAP in the operating room may not be reflective of PCWP. Figure 7 provides a comprehensive algorithm for management of RVF.

#### Early extubation

Positive pressure ventilation increases PVR and decreases CO in an RV-dependent circulation. Positive end-expiratory pressure (PEEP) and mean airway pressure should be kept at the minimum required to avoid atelectasis (223). Ultra-fast-track anesthesia, consisting of early extubation within 4 h of the surgery, is feasible with either median sternotomy or LT (224). Early extubation with spontaneous breathing and negative intrathoracic pressure minimizes RV afterload and is associated with less RVF and pneumonia, improved RVSWI and shorter length of stay (224, 225). Unfortunately, inhaled iNO therapy often delays extubation in patients at risk of RVF, and early administration of inodilators such as milrinone may hasten iNO weaning.

#### Vasoactive support

In patients undergoing LVAD surgery, the RV remains preload dependent, but with much narrower limits. At a CVP above 10–12 mmHg, volume loading further increases filling pressures without improvement in RVSWI or CO (226). Fluid responsiveness must not be mistaken for fluid tolerance, and resuscitation should be judicious (227). Inotropic support with either dobutamine, milrinone or epinephrine will improve contractility, forward flow and decrease CVP. At doses higher than 2–5 μg/kg/min, dobutamine causes a rise in PVR, and milrinone is preferred for its vasodilatory effect on the pulmonary circulation. If vasopressor support is required following milrinone initiation, low-dose vasopressin can effectively increase systemic vascular resistance (SVR) to the same extent as norepinephrine without modifying PVR (228). Intratracheal and inhaled milrinone have been successfully used to improve RVF, reduce PVR and mean PA pressures, while minimizing the effect on SVR (229, 230). Levosimendan is a calcium sensitizer that can be administered in combination with other inotropes to improve RV contractility. Reports in the LVAD population are scarce (231), but randomized trials involving levosimendan in general cardiac surgery have not shown improved outcomes (232–234). Currently there is no evidence to support levosimendan use in LVAD patients.

#### Pulmonary vasodilators

Pulmonary vasodilators may reduce RV afterload but should only be initiated once LVAD speeds are optimized. If a patient exhibits persistent pulmonary hypertension despite adequate LV offloading and a low PCWP, then pulmonary vasodilators may be of benefit. However, if the LV remains congested, pulmonary vasodilators may increase LV preload and PCWP with minimal improvement in RV afterload or performance (235).

##### Inhaled nitric oxide

Inhaled iNO is a selective pulmonary vasodilator that activates guanylate cyclase to increase cyclic guanylyl monophosphate in the smooth muscle only in vessels perfusing oxygenated alveoli, reducing intrapulmonary shunting and V/Q mismatch. Unlike intravenous inodilators such as milrinone and dobutamine, iNO has minimal effects of SVR and blood pressure, making it easier to maintain RV perfusion pressure. iNO is commonly used pre-emptively in LVAD surgery due to a favorable risk-benefit profile, with cost and time-to-extubation being the primary deterrents. Methemoglobinemia is rarely seen at clinically relevant doses of 40 ppm or less (236). Small, randomized controlled trials have demonstrated the efficacy of iNO in reducing PVR and mean PA pressure over placebo, but with a non-significant reduction in RVF, probably related to insufficient sample size and >10% crossover (237–239). The benefit of iNO seems limited to patients with elevated mean PA pressures or elevated PVR after PCWP normalization, regardless of CVP values (238–240). Abrupt discontinuation of iNO can result in rebound pulmonary hypertension and RVF. In the absence of a PAC or TEE, systemic indices of RV function such as CVP, lactate, urine output and bloodwork should be carefully monitored throughout the weaning process.

##### Inhaled prostaglandins

Inhaled prostaglandins have also been used intra-operatively and in the early post-operative setting to reduce RV afterload. Epoprostenol is a synthetic analog of prostacyclin that promotes pulmonary vasodilatation by increasing intracellular cyclic adenylyl monophosphate. Epoprostenol must be nebulized continuously within the breathing circuit to exert local selective vasodilatation. In a non-randomized trial, Epoprostenol has shown to effectively reduce mean PA pressures after LVAD support (241). Administering epoprostenol before CPB increases risk of bleeding, likely due to interference with platelet aggregation (241). Iloprost is another synthetic analog of prostacyclin with a more stable half-life that can be given intermittently via a nebulizer. Unlike epoprostenol or iNO, iloprost can be continued in patients after extubation. The addition of iloprost in LVAD patients receiving iNO has been shown to further reduce PVR, mean PA pressure and to increase TAPSE (242).

##### Sodium nitroprusside

Sodium nitroprusside is a potent arterial vasodilator with immediate effect than can reduce PVR as effectively as iNO (243). The associated decrease in SVR may be especially useful in hypertensive patients with elevated PCWP, in which increasing LVAD speeds are unable to decompress the LV (244, 245). Unlike iNO, SNP is a non-selective intravenous medication that inhibits the physiological vasoconstrictive response to hypoxia. SNP will therefore vasodilate pulmonary vessels perfusing unventilated alveoli, causing right to left shunt and hypoxemia. Cyanide toxicity can occur with prolonged SNP infusions above 2 mcg/kg/min, and serum cyanide levels should be performed routinely for the duration of the infusion.

##### Phosphodiesterase 5 inhibitors

Sildenafil is a phosphodiesterase 5 inhibitor (PDE5i) that increases cyclic guanylyl monophosphate by inhibiting its degradation, and is commonly used to wean off iNO or milrinone in patients with borderline RV function to prevent a rebound effect (246). Few studies have assessed the effect of sildenafil on clinical outcomes, but the limited data suggests a benefit for patients with persistent pulmonary hypertension after LVAD, with reduction of PVR and mPAP, and increase in RV function and CO (247). Simultaneous high-dose sildenafil, iloprost and iNO have been trialed, yielding very low rates of RVF and no need for RVAD (248). Notably, in a propensity-score-matched INTERMACS registry analysis, pre-treatment with sildenafil was associated with a paradoxical increase in RVF rates. However, this result may be due to between-group differences in baseline RV afterload, despite matching (249).

In the long-term, PDE5i could have favorable effects on RV function by decreasing RV afterload, such that it may be reasonable to try in patients with low PCWP who still display elevated PVR after LVAD. However, the major determinant of functional capacity in LVAD patients is a disproportionate rise of PCWP with activity that may limit the potential benefits of sildenafil (250). Randomized data is lacking until the results of the SOPRANO trial become available, in which macitentan will be assessed as adjunctive therapy (NCT02554903). A meta-analysis of 6 observational studies reporting data on RVF demonstrated no benefit of sildenafil to reduce RVF with very high heterogeneity between the studies included (251). These neutral results were confirmed in a propensity-matched analysis of the STS registry, and do not support the indiscriminate use of PDE5i.

#### Volume status and kidney function

The differential and management for a high CVP after separation from CPB can be challenging, reflecting a combination of volume status, RV function, hemodynamic support, respiratory function, surgical manipulation and LVAD performance. The RV is directly visible during surgery and can be regularly assessed for signs of volume overload and distention. If intraoperative TEE shows inferior vena cava (IVC) plethora, a distended RV and adequate LV filling, then diuretics may help to release RV wall tension and improve contractility. AKI is commonly seen in 25–35% patients undergoing LVAD support, and RRT is initiated in 10–15%. Both AKI and RRT are associated with early RVF and RVAD use, and higher subsequent mortality (252–254). Pre-operative predictors for AKI and RRT include an eGFR <45 mL/min/1.73 m^2^, presence of proteinuria and CVP/PCWP >0.54 (252). This association between RRT and mortality may reflect a sicker patient population, and not deleterious effects directly related to RRT. In high-risk patients, pre-emptive initiation of RRT before systemic congestion ensues may be beneficial, especially if urine output remains inadequate after high-dose diuretics. This theory was tested in a small study of 21 patients with eGFR <30 mL/min/1.73 m^2^ treated with aggressive pre-operative optimization including IABP placement and immediate RRT after weaning CPB, with similar rates of RVF and 1-year survival as patients with normal eGFR (255).

#### Heart rhythm management considerations

##### Heart rate

Temporary epicardial pacing wires are routinely implanted prior to chest closure, allowing optimization of heart rate (HR) and CO. Many patients will have permanent implanted devices, and regular interrogation can prevent competition between temporary and permanent devices. Cardiac surgery patients are routinely paced between 80 and 100 bpm in the immediate post-operative period, which maximizes output and minimizes dilatation. A higher HR may increase PCWP and thus augment RV afterload (256). If the RV has a restrictive filling pattern, CO will heavily rely on the HR, as stroke volume will plateau at low volumes. Thus, in a truly restrictive RV with adequately unloaded LV and low PCWP, higher pacing rate may result beneficial.

##### Atrial arrhythmias

Atrial fibrillation is present in 30–40% of patients at the time of implant. After LVAD surgery, half of the patients convert to sinus rhythm but an additional 10% experience *de novo* post-operative atrial fibrillation (257, 258). In both INTERMACS and EUROMACS registries, atrial fibrillation is seen in 20–25% of LVAD outpatients and is associated to decreased survival, quality of life and functional capacity, likely reflecting different baseline characteristics (259, 260). Atrial contraction at the end of diastole contributes around 15–20% to CO in normal individuals. Ventricles with restrictive filling and elevated end-diastolic pressures may be more susceptible to losing atrial contribution. In the acute setting, patients with restrictive RV filling may experience hemodynamic compromise with drop in flows due to RVF with atrial arrhythmias, and a rhythm control strategy may be advisable in these specific cases (261). An association between atrial arrhythmias and RVF and RVAD need has been reported, although the temporal sequence of events is unclear and atrial fibrillation is probably a marker of disease severity and elevated RA pressures (262).

##### Ventricular arrhythmias

Sustained ventricular arrhythmias (>30 s) occur in the early post-operative period in 20–25% of patients, most often in the first 15 days after surgery (263–265). Ventricular arrhythmias can compromise RV output and LV filling, resulting in suction events. Interventricular septum position should be assessed and suction events ruled out, especially if the ventricular arrhythmias are accompanied by reduced flows and MAP (218). Ventricular arrhythmias may triggered by metabolic derangements, R-on-T pacing, RV ischemia related to distension, or damage/occlusion of the RCA (263). Pre-operative ventricular arrhythmias are the strongest predictor for post-operative ventricular arrhythmias, highlighting the importance of scar-mediated re-entry as a leading mechanism. Initiation may be triggered by increased adrenergic tone aggravated by inotropic support. Only a minority of patients undergoing ventricular tachycardia (VT) ablation show arrhythmias related to the inflow cannula (266). RVAD at the time of LVAD insertion halved the risk of early ventricular arrhythmias after multivariate adjustment, suggesting that RVF contributes to ventricular arrhythmias in the acute setting (264).

Electrical storm is seen in 6% of patients and carries a poor prognosis with very high in-hospital mortality (264, 267, 268). Recurrent shocks can cause myocardial stunning and progressive RVF with increased support requirements including inotropes, pulmonary vasodilators, or RVAD, a functional deterioration not seen with anti-tachycardia pacing (263). Rarely, VT ablation should be considered in the acute setting for patients with RVF and recurrent intractable VT (263–265). For patients with recurrent ventricular fibrillation without preceding VT, sympatholytic therapies such as stellate ganglion blockade or ablation of the Purkinje fibers may be helpful (263, 264, 268).

##### Cardiac resynchronization therapy

Cardiac resynchronization therapy (CRT) is initiated in many patients with advanced HF patients who later undergo LVAD implant. There is no improvement in invasive hemodynamic measurements with biventricular pacing compared to no pacing or right ventricular pacing only (269–271). Similarly, there are no differences between pacing modes in LV unloading during invasive ramp tests (271). Although some patients may benefit from CRT to improve RV contractility (272), frequent pack changes, reduced exercise capacity and quality of life with biventricular pacing suggests that turning off the LV-lead is advisable for most patients after LVAD (266, 273, 274).

### Right ventricular mechanical circulatory support

#### Timing of right ventricular assist device implantation

Right ventricular assist device implantation is more effective as a pre-emptive strategy than a rescue device. The pre-operative LVAD assessment should identify patients at moderate to high risk of RVF. Ideally, the decision to implement RVAD support is made at the time of LVAD implantation, and not after multi-organ failure secondary to congestion and hypoperfusion are established. Several studies suggest improved survival and end-organ preservation with planned RVAD support compared to rescue (275, 276). 30-day survival for planned, combined LVAD + RVAD implant parallels survival of patients who require LVAD support alone (277). This trend is further evidenced by a STS registry analysis which showed that mortality post-operative rescue RVAD increased by almost 50% when compared to pre-emptive, planned RVAD + LVAD simultaneous implantation (278). Thus, liberal use of RVAD in patients at high-risk for RVF may be beneficial (279).

#### Extracorporeal membrane oxygenation vs. right ventricular assist device

Venoarterial (VA) ECMO reduces RV preload by diverting blood from the venous system through an external pump and oxygenator, which then returns to the arterial circulation through direct aortic cannulation in the ascending aorta or through a femoral artery if peripherally cannulated. Optimizing the fine balance between the ECMO circuit and the LVAD can be challenging. If the ECMO speed is too high, there may be inadequate flow through the RV and pulmonary circulation, leading to LV underfilling and suction events. Conversely, if the LVAD speeds is too low, the increased LV afterload may lead to inadequate LV decompression with secondary pulmonary congestion. Despite these challenges, V-A ECMO has been used successfully for acute RVF following LVAD implant, but with well-recognized risks (280, 281). In an adjusted analysis using data from the STS registry, LVAD patients who required ECMO support showed higher rates of acute limb ischemia, reoperation, pneumonia, wound infection, and mortality compared to those who received an RVAD, (54.2% vs. 40.5%, *p* < 0.001) (278). Notably, peripheral V-A ECMO significantly increases LV afterload and should not be used in patients receiving LVAD as bridge to recovery.

#### Types of right ventricular assist device

Right ventricular assist device support can be provided through percutaneous or central cannulation. In recent years, percutaneous RVAD has gained popularity over central RVAD for its minimal invasiveness, but the preference is supported by little evidence. Disadvantages of central RVAD include the need for a second surgery if not implanted at the time of LVAD, and a third for its removal, although percutaneous removal is possible if planned at the time of insertion (277). Although large scale studies are lacking, percutaneous RVADs are associated with a shorter length of stay, shorter time on mechanical ventilation and less blood product utilization with a trend toward lower rates of RRT (27.3% vs. 52.4%; *p* = 0.09) and mortality (21.1% vs. 42.9%; *p* = 0.14) (282). An oxygenator allows for correction of hypoxemia and can be connected to central RVADs and some percutaneous models. A comparison between RVAD with or without oxygenator showed earlier acidosis resolution and further decrease in vasoactive medication with shorter time on support if an oxygenator was added to the system (283).

The most commonly used central RVAD device is the Levitronix CentriMag^®^ (Abbott, IL, USA), with an inflow cannula in the RA, and an outflow cannula in the PA. The inflow cannula can be surgically implanted, or peripherally inserted through the femoral vein. CentriMag^®^ RVAD can provide up to 10 L/min of support, and an oxygenator can be incorporated to the circuit if there is concomitant lung disease with associated hypoxemia (284).

Percutaneous RVAD systems currently available include the TandemHeart^®^ (LivaNova, London, UK), ProtekDuo^®^ (LivaNova, London, UK) and Impella RP^®^ (Abiomed, Danvers, MA, USA). Tandem Heart femoro-femoral cannulation with inflow in the superior vena cava–RA junction with a percutaneously placed cannula in the PA has been largely abandoned since the commercialization of the ProtekDuo^®^ cannula in 2014 (284). The ProtekDuo^®^ cannula is a dual lumen cannula allowing for percutaneous, single-vessel jugular venous access. The ProtekDuo^®^ cannula is placed under echocardiographic or fluoroscopic guidance with a proximal port in the RA and a distal port in the main PA. The cannula is connected to an extracorporeal centrifugal pump [either Lifesparc^®^ (LivaNova, London, UK), ECMO or CentriMag^®^ console], able to provide flow up to 4–4.5 L/min (284), and allows for the connection of an oxygenator. The ProtekDuo^®^ has been successfully used in LVAD patients and is approved for up to 30 days of use (285–287). Its jugular insertion allows for patient ambulation and better rehabilitation, but can also cause superior vena cava syndrome given the large cannula size (288). Specific to the shape of Protek Duo is RCA compression caused by the bending of the cannula within the RV (289).

The Impella RP^®^ is a dual-lumen 22 Fr cannula inserted under fluoroscopy through the femoral vein that incorporates a microaxial pump to propel blood from the IVC into the PA. Unlike the ProtekDuo^®^, the Impella RP^®^ cannot accommodate an oxygenator, and should not be used if hypoxemia is a concern, as an upgrade to surgical RVAD may be necessary in that scenario. It has been used successfully in RVF after LVAD and can provide up to 4–4.5 L/min of flow, but it is only approved for less than 14 days of support (287, 290), and its femoral placement precludes ambulation. Complications that can occur are device migration, clotting of the cannula or of the purge system and hemolysis, due to the high speed required by the microaxial pump (usually above 30,000 rpm to provide full support) (290).

Contraindications to use percutaneous RVAD are mechanical valves, pulmonary or supravalvular stenosis, severe pulmonic insufficiency, or clots in the right chambers, but a surgical RVAD with the inflow cannula in the RA or VA-ECMO could be used if the clot is limited to the RV. Impella RP should be used with caution if there are IVC filters or deep vein thrombosis. Common to both percutaneous RVADs is the risk of tricuspid or pulmonary valve damage, but incidence is low (290). Also, fracture of the cannula, bleeding at the entry site and PA perforation can occur with both percutaneous devices (290, 291). RVAD flows should be kept below LVAD flow to avoid pulmonary edema and hemorrhage, which have been associated with surgical RVAD flows >4 L/min (292).

#### Right ventricular assist device weaning

There are no standardized protocols for weaning RVAD support. In preparation for decannulation, an RVAD ramp study should be performed under echocardiographic guidance with advanced hemodynamic monitoring to assess RV response to decrements in speed, although simultaneous PAC insertion may not be possible in many cases while on RVAD support. At each speed, CVP, MAP, LVAD flow and the occurrence of suction events should be assessed, along with TAPSE, tricuspid s′ and the velocity-time integral in the RV outflow tract. If hemodynamics remain unchanged following a reduction in 0.5–1 L/min, further reductions are attempted as tolerated. Risk of thrombosis increases at flows below 2 L/min, and anticoagulation should be initiated, targeting an activated clotting time (ACT) >160 s, or >200 s if an oxygenator is attached to the pump to prevent clotting. In some cases, it may be preferable to maintain a low flow on the RVAD for a more prolonged time while on proper anticoagulation and assess urine output and lactate before proceeding to next steps. If CVP remains unchanged and LVAD flow is stable with no suction events, full anticoagulation is administered (ACT >300), and RVAD flows dropped to 1 L/min for 5–10 min. If all parameters remain stable and the RV is functioning adequately on TEE, the circuit can be clamped in preparation for decannulation (293).

#### Exit strategies: BerlinHeart excor right ventricular assist device

If weaning cannot be achieved after multiple attempts, an exit strategy is required, as percutaneous RVADs are not approved for long-term use. Unfortunately, in this scenario, prognosis is poor regardless of strategy. In patients who have recovered end-organ function, OHT candidacy needs to be reassessed. If the patient can be listed for OHT but long wait times are expected, more durable biventricular support can be achieved with the Excor RVAD^®^ (BerlinHeart, Berlin, Germany). Excor is a paracorporeal pulsatile mechanical ventricle of different sizes that can be used exclusively as RVAD or combined with an LVAD. A common Excor^®^ complications include thrombosis of the mechanical ventricle, which is risky on the right side, high rates of bleeding and wound infections (294, 295). However, approximately 50% of carefully selected patients receiving an upgrade to Excor support following a failed temporary RVAD wean could be successfully bridged to OHT, with similar survival after OHT as other recipients (294–296).

#### Exit strategies: Durable right ventricular assist devices

The use of durable BIVAD remains highly experimental and should only be used as a last resort in critically ill patients as BTT candidacy. Most published cases of durable intracorporeal RVAD using bilateral continuous-flow VADs underwent upfront BIVAD and not staged implantation after RVF, which has been associated with worse outcomes (297). However, patients receiving durable BIVAD support were more acutely ill than those receiving isolated LVAD (298). When performed simultaneously, durable BIVAD is as effective as total artificial heart regarding survival until transplantation. Durable BIVAD is associated with longer time on support but higher rates of hospital discharge (299). Most durable BIVAD strategies involve off-label use of the Medtronic HVAD^®^ pump, a smaller centrifugal, continuous-flow device that permitted intrathoracic placement (300, 301). 1-year survival with a HVAD-BIVAD configuration was 56% is a multi-center collaboration (302). Similar survival rates have been described with either right atrial or right ventricular inflow cannula configurations, with pump thrombosis occurring in 30% of cases (297–299, 302, 303). Unfortunately, the Medtronic HVAD was withdrawn from the worldwide market in 2021 amidst studies demonstrating higher rates of neurological events and mortality. As an alternative, dual HM3 support has been demonstrated, but necessitates either partial cardiotomy (304–306) or right atrial configuration (307, 308). Comprehensive studies are lacking, as reported survival rates with HM3 in an RVAD configuration widely ranges from 30% at 3 months to 92% at 18 months (298, 306–308).

## Late right ventricular failure

Late RVF is currently defined as RVF occurring at least 30 days after the implant, as recommended by the MCS-ARC (Figure 1). The prevalence of moderate-severe late RVF is 20% at 1-month, decreasing to 3–5% at 3 months and remaining stable thereafter (309). However, if milder cases are included, the prevalence of late RVF are estimated to be as high as 40% (310). In patients with an LVAD as BTT, late RVF is associated with higher urgent HT rates (309). Although the data are conflicting (280), late RVF is a major risk factor for primary graft dysfunction, in-hospital mortality, and high 1 and 5-year mortality after HT (22, 311). RV assessment is usually more permissive and LVAD indication is more liberal in the BTT population as the time on support is expected to be shorter, but these results highlight that careful RV assessment and aggressive RVF treatment should be pursued to improve outcomes after HT.

Late RVF is associated with lower hemoglobin, higher gastrointestinal bleeding and stroke rates, VAD-related and non-VAD-related infections and persistent kidney impairment during follow-up (39, 309, 312, 313). Quality of life and functional capacity is also decreased in patients with late RVF (309, 313). The pathophysiology underlying the non-cardiovascular complications of RVF is poorly defined. LVAD patients have an abnormal von Willebrand factor metabolism and overexpression of angiopoetin-2 that increases the risk of arteriovenous malformations and creates a fragile vasculature. Along with lack of pulsatility, RVF causes malnutrition due to gut edema and hepatic congestion, venous hypertension, blood stasis, coagulopathy and increased intravascular pressure within these abnormal vessels that predispose them to rupture and bleeding in the GI tract or brain (204). In addition to hemorrhagic complications, the extravasated blood and blood stasis act as an ideal substrate for infection (204).

The response of the RV to LVAD will be patient-specific depending on interventricular interdependence and RV-PA coupling. In general, higher LVAD speeds will worsen RV systolic function but improve RV compliance and relaxation (314, 315). In most cases, the RV remains sensitive to afterload (126) and higher speeds will provide better unloading, reflected in the improved RV performance and fewer RVF and HF admissions observed at higher speeds (316–319). Pressure-volume loops before and after LVAD implantation demonstrate that despite higher CVP for a similar Ea, the stroke volume/end-systolic volume as a measure of RV-PA coupling significantly improved, suggesting better RV efficiency (320). The elevated CVP may therefore reflect diastolic rather than systolic dysfunction, explaining the better long-term performance of the RV at higher speeds (321, 322). The association between recurrent HF admissions, impaired RV relaxation and restrictive RV filling is demonstrated by higher RVF rates in patients with a deep CVP Y descent or prolonged diastolic plateau (>55% of the diastole) (323, 324).

A detailed description of long-term management of RVF in LVAD patients is beyond the scope of this review, but it is worth mentioning that invasive ramp studies are feasible and reliable as soon as 1–3 months after the implant (317) and allow case-by-case pump speed optimization. Echocardiographic surveillance is mandatory during follow-up, and can be useful in estimating filling pressures (325). With limited evidence, it seems that optimization of HF medication may improve LV function, thereby reducing RV afterload and improving its function (326). In the setting of late RVF, *de novo* AR should be ruled out, and revision of the log files looking for a progressive increase in power for a similar flow can help in suspecting an outflow graft obstruction. If the LVAD speed is optimized and there are no lesions amenable to repair, RVF should be managed as per the current HF guidelines with diuretic adjustment, or dialysis (hemodialysis or peritoneal) if RVF is refractory to medical management (327–329). Inotropes may be an option for these patients (330), as the use of oral milrinone or intermittent levosimendan infusions have been successfully reported (331, 332). RVADs for late RVF should be restricted to patients who remain on the OHT list and develop refractory RVF with cardiogenic shock.

## Conclusion

Right ventricular failure is a devastating complication occurring after LVAD implantation, but it can be predicted pre-operatively in the basis of clinical features and a combination of echocardiographic and hemodynamic RV metrics here summarized. Intra-operative assessment of RV function is paramount to decide on early RVAD support to improve patient survival. This review offers a comprehensive guidance to provide the best supportive treatment for the patient with RVF after LVAD in the early post-operative phase and considerations about RV performance later during the outpatient follow up.

## Author contributions

ER-A and DB generated the figures. All authors contributed to the writing and editing of the manuscript.

## Glossary

LVAD: left ventricular assist device
HF: heart failure
DT: destination therapy
BTT: bridge to transplant
OHT: orthotopic heart transplant
RV: right ventricle
RVF: right ventricular failure
RVAD: right ventricular assist device
HRAEs: hemocompatibility-related adverse events
INTERMACS: interagency registry of mechanical circulatory support
CVP: central venous pressure
MCS-ARC: mechanical circulatory support–academic research consortium
BSA: body surface area
HM2: Heart Mate II
HM3: Heart Mate III
LVEDD: left ventricular end-diastolic diameter
TR: tricuspid regurgitation
LA: left atrium
BMI: body mass index
eGFR: estimated glomerular filtration rate
CKD: chronic kidney disease
BUN: blood urea nitrogen
NGAL: neutrophil gelatinase-associated lipocalin
RRT: renal replacement therapy
INR: international normalized ratio
ECMO: extracorporeal membrane oxygenation
CCR: chemokine receptor
IL: interleukin
PVR: pulmonary vascular resistance
VE: ventilatory efficiency
VCO2: carbon dioxide output
VO2: oxygen consumption
PAPi: pulmonary artery pulsatility index
PA: pulmonary artery
NICM: non-ischemic cardiomyopathy
TAPSE: tricuspid annular plane systolic excursion
RA: right atrium
AUC: area under the curve
TPG: transpulmonary gradient
PCWP: pulmonary capillary wedge pressure
TPDG: transpulmonary diastolic gradient
CO: cardiac output
Ea: arterial elastance
SVI: stroke volume index
RVSWI: right ventricular stroke work index
mPAP: mean pulmonary artery pressure
MAP: mean arterial pressure
PAC: pulmonary artery catheter
Ees: end-systolic elastance
CPB: cardiopulmonary bypass
FDA: food and drug administration
ROTEM: rotational thromboelastometry
ICU: intensive care unit
TV: tricuspid valve
MR: mitral regurgitation
AR: aortic regurgitation
TAVI: transcatheter aortic valve implantation
RCA: right coronary artery
TTE: transthoracic echocardiography
TEE: transesophageal echocardiography
PEEP: positive end-expiratory pressure
iNO: nitric oxide
SVR: systemic vascular resistance
SNP: sodium nitroprusside
PDE5i: phosphodiesterase 5 inhibitor
AKI: acute kidney injury
IVC: inferior vena cava
HR: heart rate
VT: ventricular tachycardia
CRT: cardiac resynchronization therapy
LV: left ventricle
VA: venoarterial
ACT: activated clotting time
BIVAD: biventricular assist device

## References

[1] Goldstein DJ Meyns B Xie R Cowger J Pettit S Nakatani T Third annual report from the ISHLT mechanically assisted circulatory support registry: a comparison of centrifugal and axial continuous-flow left ventricular assist devices. *J Heart Lung Transplant.* (2019) 38:352–63. 10.1016/j.healun.2019.02.004 30945637

[2] Frankfurter C Molinero M Vishram-Nielsen JKK Foroutan F Mak S Rao V Predicting the risk of right ventricular failure in patients undergoing left ventricular assist device implantation: a systematic review. *Circ Heart Fail.* (2020) 13:e006994. 10.1161/CIRCHEARTFAILURE.120.006994 32981331

[3] Molina EJ Shah P Kiernan MS Cornwell WK III Copeland H Takeda K The society of thoracic surgeons intermacs 2020 annual report. *Ann Thorac Surg.* (2021) 111:778–92. 10.1016/j.athoracsur.2020.12.038 33465365

[4] de By TMMH Schoenrath F Veen KM Mohacsi P Stein J Alkhamees KMM The European Registry for Patients with mechanical circulatory support of the European Association for Cardio-Thoracic Surgery: third report. *Eur J Cardiothorac Surg.* (2022) 62:ezac032. 10.1093/ejcts/ezac350 35150247

[5] Hall SA Copeland H Alam A Joseph SM. The “right” definition for post-left ventricular assist device right heart failure: the more we learn, the less we know. *Front Cardiovasc Med.* (2022) 9:893327. 10.3389/fcvm.2022.893327 35557521 PMC9087190

[6] Cruz Rodriguez JB Stewart GC Pamboukian SV Tallaj JA Rajapreyar I Kirklin JK Clinical characteristics and outcomes of patients requiring prolonged inotropes after left ventricular assist device implantation. *Artif Organs.* (2020) 44:E382–93. 10.1111/aor.13692 32242954

[7] Muslem R Ong CS Tomashitis B Schultz J Ramu B Craig ML Pulmonary arterial elastance and INTERMACS-defined right heart failure following left ventricular assist device. *Circ Heart Fail.* (2019) 12:e005923. 10.1161/CIRCHEARTFAILURE.119.005923 31401840

[8] Grandin EW Troutman GS Gulati AA Zamani P Mazurek JA Atluri P A modified grading system for early right heart failure matches functional outcomes and survival after left ventricular assist devices. *ASAIO J.* (2021) 67:185–91. 10.1097/MAT.0000000000001203 32618585

[9] LaRue SJ Raymer DS Pierce BR Nassif ME Sparrow CT Vader JM. Clinical outcomes associated with INTERMACS-defined right heart failure after left ventricular assist device implantation. *J Heart Lung Transplant.* (2017) 36:475–7. 10.1016/j.healun.2016.12.017 28238616 PMC8272542

[10] Kormos RL Antonides CFJ Goldstein DJ Cowger JA Starling RC Kirklin JK Updated definitions of adverse events for trials and registries of mechanical circulatory support: a consensus statement of the mechanical circulatory support academic research consortium. *J Heart Lung Transplant.* (2020) 39:735–50. 10.1016/j.healun.2020.03.010 32386998

[11] Mehra MR Uriel N Naka Y Cleveland JCJ Yuzefpolskaya M Salerno CT A fully magnetically levitated left ventricular assist device – Final report. *N Engl J Med.* (2019) 380:1618–27. 30883052 10.1056/NEJMoa1900486

[12] Lee S Katz JN Jorde UP Moazami N John R Sundareswaran KS Outcomes of adult patients with small body size supported with a continuous-flow left ventricular assist device. *ASAIO J.* (2016) 62:646–51. 10.1097/MAT.0000000000000430 27556150 PMC5098461

[13] Ono M Sawa Y Nakatani T Tominaga R Matsui Y Yamazaki K Japanese multicenter outcomes with the HeartMate II left ventricular assist device in patients with small body surface area. *Circ J.* (2016) 80:1931–6. 10.1253/circj.CJ-16-0203 27373233

[14] Molina E Jain A Ahmed S Lam P Rao S Hockstein M The impact of left ventricular size on outcomes after centrifugal-flow left ventricular assist device implantation. *Eur J Cardiothorac Surg.* (2021). 10.1093/ejcts/ezab480 [Epub ahead of print]. 34788417

[15] Zafar F Villa CR Morales DL Blume ED Rosenthal DN Kirklin JK Does small size matter with continuous flow devices?: analysis of the INTERMACS database of adults with BSA =1.5 m. *JACC Heart Fail.* (2017) 5:123–31. 10.1016/j.jchf.2016.09.009 27816511

[16] Shah P Birk S Maltais S Stulak J Elmi A Pagani FD Left ventricular assist device outcomes based on flow configuration and pre-operative left ventricular dimension: an interagency registry for mechanically assisted circulatory support analysis. *J Heart Lung Transplant.* (2017) 36:640–9. 10.1016/j.healun.2016.12.004 28087105

[17] Kawabori M Kurihara C Conyer R Sugiura T Critsinelis AC Lee V-V A left ventricular end-diastolic dimension less than 6.0 cm is associated with mortality after implantation of an axial-flow pump. *J Thorac Cardiovasc Surg.* (2019) 157:2302–10. 10.1016/j.jtcvs.2019.01.015 30797583

[18] Anne Dual S Nayak A Hu Y Schmid Daners M Morris AA Cowger J. Does size matter for female continuous-flow LVAD recipients? A translational approach to a decade long question. *ASAIO J.* (2022) 68:21–7. 10.1097/MAT.0000000000001443 34156789

[19] Kiamanesh O Rankin K Billia F Badiwala MV. Left ventricular assist device with a left atrial inflow cannula for hypertrophic cardiomyopathy. *JACC Case Rep.* (2020) 2:2090–4. 10.1016/j.jaccas.2020.10.006 34317114 PMC8299761

[20] Khan MS Yuzefpolskaya M Memon MM Usman MS Yamani N Garan AR Outcomes associated with obesity in patients undergoing left ventricular assist device implantation: a systematic review and meta-analysis. *ASAIO J.* (2020) 66:401–8. 10.1097/MAT.0000000000001019 31192852

[21] Han J Mauro CM Kurlansky PA Fukuhara S Yuzefpolskaya M Topkara VK Impact of obesity on readmission in patients with left ventricular assist devices. *Ann Thorac Surg.* (2018) 105:1192–8. 10.1016/j.athoracsur.2017.10.043 29397927

[22] Takeda K Takayama H Colombo PC Jorde UP Yuzefpolskaya M Fukuhara S Late right heart failure during support with continuous-flow left ventricular assist devices adversely affects post-transplant outcome. *J Heart Lung Transplant.* (2015) 34:667–74. 10.1016/j.healun.2014.10.005 25577566

[23] Ebong IA Goff DCJ Rodriguez CJ Chen H Bertoni AG. Mechanisms of heart failure in obesity. *Obes Res Clin Pract.* (2014) 8:e540–8. 10.1016/j.orcp.2013.12.005 25434909 PMC4250935

[24] Saito A Amiya E Hatano M Shiraishi Y Nitta D Minatsuki S Controlling nutritional status score as a predictive marker for patients with implantable left ventricular assist device. *ASAIO J.* (2020) 66:166–72. 10.1097/MAT.0000000000000972 30913100

[25] Yost G Tatooles A Bhat G. Preoperative nutritional assessment with the prognostic nutrition index in patients undergoing left ventricular assist device implantation. *ASAIO J.* (2018) 64:52–5. 10.1097/MAT.0000000000000625 28692526

[26] Critsinelis AC Kurihara C Kawabori M Sugiura T Civitello AB Morgan JA. Preoperative prealbumin level as a predictor of outcomes in patients who underwent left ventricular assist device implantation. *Am J Cardiol.* (2017) 120:1998–2002. 10.1016/j.amjcard.2017.08.004 28958451

[27] Uribarri A Rojas SV Hanke JS Dogan G Siemeni T Kaufeld T Prognostic value of the nutritional risk index in candidates for continuous flow left ventricular assist device therapy. *Rev Esp Cardiol.* (2019) 72:608–15. 10.1016/j.rec.2018.05.029 30078744

[28] Davis BH Boehme AK Pamboukian SV Allon M George JF Dillon C Improvement in kidney function after ventricular assist device implantation and its influence on thromboembolism, hemorrhage, and mortality. *ASAIO J.* (2020) 66:268–76. 10.1097/MAT.0000000000000989 30883405 PMC6744354

[29] Mohamedali B Bhat G. The influence of pre-left ventricular assist device (LVAD) implantation glomerular filtration rate on long-term LVAD outcomes. *Heart Lung Circ.* (2017) 26:1216–23. 10.1016/j.hlc.2017.01.002 28342643

[30] Tang W Verbrugge F Mullens W. *Cardiorenal Syndrome in Heart Failure*. Cham: Springer (2020). 10.1007/978-3-030-21033-5

[31] Hasin T Topilsky Y Schirger JA Li Z Zhao Y Boilson BA Changes in renal function after implantation of continuous-flow left ventricular assist devices. *J Am Coll Cardiol.* (2012) 59:26–36. 10.1016/j.jacc.2011.09.038 22192665

[32] Kilic A Chen CW Gaffey AC Wald JW Acker MA Atluri P. Preoperative renal dysfunction does not affect outcomes of left ventricular assist device implantation. *J Thorac Cardiovasc Surg.* (2018) 156:1093–101.e1. 10.1016/j.jtcvs.2017.12.044 30017440

[33] Testani JM Cappola TP Brensinger CM Shannon RP Kimmel SE. Interaction between loop diuretic-associated mortality and blood urea nitrogen concentration in chronic heart failure. *J Am Coll Cardiol.* (2011) 58:375–82. 10.1016/j.jacc.2011.01.052 21757114 PMC3980479

[34] Ruiz-Cano MJ Morshuis M Koster A Lauenroth V Prashovikj E Gummert J Risk factors of early right ventricular failure in patients undergoing LVAD implantation with intermediate intermacs profile for advanced heart failure. *J Card Surg.* (2020) 35:1832–9. 10.1111/jocs.14696 32557925

[35] Pinsino A Mondellini GM Royzman EA Hoffman KL D’Angelo D Mabasa M Cystatin C- versus creatinine-based assessment of renal function and prediction of early outcomes among patients with a left ventricular assist device. *Circ Heart Fail.* (2020) 13:e006326. 10.1161/CIRCHEARTFAILURE.119.006326 31959016

[36] Pronschinske KB Qiu S Wu C Kato TS Khawaja T Takayama H Neutrophil gelatinase-associated lipocalin and cystatin C for the prediction of clinical events in patients with advanced heart failure and after ventricular assist device placement. *J Heart Lung Transplant.* (2014) 33:1215–22. 10.1016/j.healun.2014.06.007 25049066 PMC4252882

[37] Topkara VK Coromilas EJ Garan AR Li RC Castagna F Jennings DL Preoperative proteinuria and reduced glomerular filtration rate predicts renal replacement therapy in patients supported with continuous-flow left ventricular assist devices. *Circ Heart Fail.* (2016) 9:e002897. 10.1161/CIRCHEARTFAILURE.115.002897 27932533

[38] Muslem R Caliskan K Akin S Sharma K Gilotra NA Brugts JJ Pre-operative proteinuria in left ventricular assist devices and clinical outcome. *J Heart Lung Transplant.* (2018) 37:124–30. 10.1016/j.healun.2017.07.011 28781011

[39] Yoshioka D Takayama H Colombo PC Yuzefpolskaya M Garan AR Topkara VK Changes in end-organ function in patients with prolonged continuous-flow left ventricular assist device support. *Ann Thorac Surg.* (2017) 103:717–24. 10.1016/j.athoracsur.2016.12.018 28168962

[40] Russell SD Rogers JG Milano CA Dyke DB Pagani FD Aranda JM Renal and hepatic function improve in advanced heart failure patients during continuous-flow support with the HeartMate II left ventricular assist device. *Circulation.* (2009) 120:2352–7. 10.1161/CIRCULATIONAHA.108.814863 19933938

[41] Critsinelis A Kurihara C Volkovicher N Kawabori M Sugiura T Manon M II Model of end-stage liver disease-excluding international normalized ratio (MELD-XI) scoring system to predict outcomes in patients who undergo left ventricular assist device implantation. *Ann Thorac Surg.* (2018) 106:513–9. 10.1016/j.athoracsur.2018.02.082 29626453

[42] Matthews JC Pagani FD Haft JW Koelling TM Naftel DC Aaronson KD. Model for end-stage liver disease score predicts left ventricular assist device operative transfusion requirements, morbidity, and mortality. *Circulation.* (2010) 121:214–20. 10.1161/CIRCULATIONAHA.108.838656 20048215 PMC2824259

[43] Yang JA Kato TS Shulman BP Takayama H Farr M Jorde UP Liver dysfunction as a predictor of outcomes in patients with advanced heart failure requiring ventricular assist device support: use of the Model of End-Stage Liver Disease (MELD) and MELD eXcluding INR (MELD-XI) scoring system. *J Heart Lung Transplant.* (2012) 31:601–10. 10.1016/j.healun.2012.02.027 22458997 PMC3358456

[44] Yalcin YC Muslem R Veen KM Soliman OI Manintveld OC Darwish Murad S Impact of preoperative liver dysfunction on outcomes in patients with left ventricular assist devices. *Eur J Cardiothorac Surg.* (2020) 57:920–8. 10.1093/ejcts/ezz337 31828334

[45] Yost GL Coyle L Bhat G Tatooles AJ. Model for end-stage liver disease predicts right ventricular failure in patients with left ventricular assist devices. *J Artif Organs.* (2016) 19:21–8. 10.1007/s10047-015-0853-x 26187243

[46] Bellavia D Iacovoni A Scardulla C Moja L Pilato M Kushwaha SS Prediction of right ventricular failure after ventricular assist device implant: systematic review and meta-analysis of observational studies. *Eur J Heart Fail.* (2017) 19:926–46. 10.1002/ejhf.733 28371221

[47] Demirozu ZT Hernandez R Mallidi HR Singh SK Radovancevic R Segura AM HeartMate II left ventricular assist device implantation in patients with advanced hepatic dysfunction. *J Card Surg.* (2014) 29:419–23. 10.1111/jocs.12318 24641429

[48] Sargent JE Dardas TF Smith JW Pal JD Cheng RK Masri SC Periportal fibrosis without cirrhosis does not affect outcomes after continuous flow ventricular assist device implantation. *J Thorac Cardiovasc Surg.* (2016) 151:230–5. 10.1016/j.jtcvs.2015.08.073 26421983

[49] Kashiyama N Toda K Nakamura T Miyagawa S Nishi H Yoshikawa Y Evaluation of right ventricular function using liver stiffness in patients with left ventricular assist device. *Eur J Cardiothorac Surg.* (2017) 51:715–21. 10.1093/ejcts/ezw419 28380632 PMC5400022

[50] Hernandez-Montfort JA Xie R Ton VK Meyns B Nakatani T Yanase M Longitudinal impact of temporary mechanical circulatory support on durable ventricular assist device outcomes: an IMACS registry propensity matched analysis. *J Heart Lung Transplant.* (2020) 39:145–56. 10.1016/j.healun.2019.11.009 31866174

[51] Shah P Pagani FD Desai SS Rongione AJ Maltais S Haglund NA Outcomes of patients receiving temporary circulatory support before durable ventricular assist device. *Ann Thorac Surg.* (2017) 103:106–12. 10.1016/j.athoracsur.2016.06.002 27577033

[52] DeFilippis EM Clerkin K Truby LK Francke M Fried J Masoumi A ECMO as a bridge to left ventricular assist device or heart transplantation. *JACC Heart Fail.* (2021) 9:281–9. 10.1016/j.jchf.2020.12.012 33714743

[53] Bertaina M Galluzzo A Rossello X Sbarra P Petitti E Prever SB Prognostic implications of pulmonary artery catheter monitoring in patients with cardiogenic shock: a systematic review and meta-analysis of observational studies. *J Crit Care.* (2022) 69:154024. 10.1016/j.jcrc.2022.154024 35344825

[54] Benjamin MM Sundararajan S Sulaiman S Miles B Walker RJ Durham L Association of preoperative duration of inotropy on prevalence of right ventricular failure following LVAD implantation. *ESC Heart Fail.* (2020) 7:1949–55. 10.1002/ehf2.12791 32526807 PMC7373884

[55] Drakos SG Janicki L Horne BD Kfoury AG Reid BB Clayson S Risk factors predictive of right ventricular failure after left ventricular assist device implantation. *Am J Cardiol.* (2010) 105:1030–5. 10.1016/j.amjcard.2009.11.026 20346326

[56] Kormos RL Teuteberg JJ Pagani FD Russell SD John R Miller LW Right ventricular failure in patients with the HeartMate II continuous-flow left ventricular assist device: incidence, risk factors, and effect on outcomes. *J Thorac Cardiovasc Surg.* (2010) 139:1316–24. 10.1016/j.jtcvs.2009.11.020 20132950

[57] Atluri P Goldstone AB Fairman AS MacArthur JW Shudo Y Cohen JE Predicting right ventricular failure in the modern, continuous flow left ventricular assist device era. *Ann Thorac Surg.* (2013) 96:854–7. 10.1016/j.athoracsur.2013.03.099 23791165 PMC4111251

[58] Janssen E Jukema JW Beeres SLMA Schalij MJ Tops LF. Prognostic value of natriuretic peptides for all-cause mortality, right ventricular failure, major adverse events, and myocardial recovery in advanced heart failure patients receiving a left ventricular assist device: a systematic review. *Front Cardiovasc Med.* (2021) 8:699492. 10.3389/fcvm.2021.699492 34307507 PMC8292668

[59] Kato TS Chokshi A Singh P Khawaja T Iwata S Homma S Markers of extracellular matrix turnover and the development of right ventricular failure after ventricular assist device implantation in patients with advanced heart failure. *J Heart Lung Transplant.* (2012) 31:37–45. 10.1016/j.healun.2011.10.007 22071239

[60] Schipper MEI Scheenstra MR van Kuik J van Wichen DF van der Weide P Dullens HFJ Osteopontin: a potential biomarker for heart failure and reverse remodeling after left ventricular assist device support. *J Heart Lung Transplant.* (2011) 30:805–10. 10.1016/j.healun.2011.03.015 21531579

[61] Tie H Welp H Martens S Seiler M Albers P Mueller K-M Impact of cardiac fibrosis and collagens on right ventricular failure and acute kidney injury in patients after continuous-flow left ventricular assist devices. *Interact Cardiovasc Thorac Surg.* (2021) 33:969–77. 10.1093/icvts/ivab180 34252191 PMC8923418

[62] Tang PC Haft JW Romano MA Bitar A Hasan R Palardy M Right ventricular failure following left ventricular assist device implantation is associated with a preoperative pro-inflammatory response. *J Cardiothorac Surg.* (2019) 14:80. 10.1186/s13019-019-0895-x 31023326 PMC6482580

[63] Nayak A Neill C Kormos RL Lagazzi L Halder I McTiernan C Chemokine receptor patterns and right heart failure in mechanical circulatory support. *J Heart Lung Transplant.* (2017) 36:657–65. 10.1016/j.healun.2016.12.007 28209402 PMC5446283

[64] Hennig F Stepanenko AV Lehmkuhl HB Kukucka M Dandel M Krabatsch T Neurohumoral and inflammatory markers for prediction of right ventricular failure after implantation of a left ventricular assist device. *Gen Thorac Cardiovasc Surg.* (2011) 59:19–24. 10.1007/s11748-010-0669-9 21225395

[65] Caruso R Verde A Cabiati M Milazzo F Boroni C Del Ry S Association of pre-operative interleukin-6 levels with interagency registry for mechanically assisted circulatory support profiles and intensive care unit stay in left ventricular assist device patients. *J Heart Lung Transplant.* (2012) 31:625–33. 10.1016/j.healun.2012.02.006 22386451

[66] Caruso R Botta L Verde A Milazzo F Vecchi I Trivella MG Relationship between pre-implant interleukin-6 levels, inflammatory response, and early outcome in patients supported by left ventricular assist device: a prospective study. *PLoS One.* (2014) 9:e90802. 10.1371/journal.pone.0090802 24594915 PMC3942482

[67] Ebner B Grant JK Vincent L Maning J Olarte N Olorunfemi O Evaluating the impact of chronic obstructive pulmonary disease on in-hospital outcomes following left ventricular assist device implantation. *J Card Surg.* (2020) 35:3374–80. 10.1111/jocs.15084 33001502

[68] Agostoni P Contini M Vignati C Del Torto A De Vecchi Lajolo G Salvioni E Acute increase of cardiac output reduces central sleep apneas in heart failure patients. *J Am Coll Cardiol.* (2015) 66:2571–2. 10.1016/j.jacc.2015.09.074 26653634

[69] Schaffer SA Bercovitch RS Ross HJ Rao V. Central sleep apnea interfering with adequate left ventricular filling in a patient with left ventricular assist device. *J Clin sleep Med.* (2013) 9:161–2. 10.5664/jcsm.2416 23372470 PMC3544385

[70] Tsuji M Amiya E Bujo C Hara T Saito A Minatsuki S Carbon monoxide diffusing capacity predicts cardiac readmission in patients undergoing left ventricular assist device implantation in Japan. *ASAIO J.* (2021) 67:1111–8. 10.1097/MAT.0000000000001363 33470633

[71] Grinstein J Sawalha Y Medvedofsky DA Ahmad S Hofmeyer M Rodrigo M VE/VCO2 slope predicts RV dysfunction and mortality after left ventricular assist device: a fresh look at cardiopulmonary stress testing for prognostication. *J Artif Organs.* (2021) 24:425–32. 10.1007/s10047-021-01261-9 33792816

[72] Løgstrup BB Nemec P Schoenrath F Gummert J Pya Y Potapov E Heart failure etiology and risk of right heart failure in adult left ventricular assist device support: the European Registry for Patients with Mechanical Circulatory Support (EUROMACS). *Scand Cardiovasc J.* (2020) 54:306–14. 10.1080/14017431.2020.1781239 32552049

[73] Oliveira GH Dupont M Naftel D Myers SL Yuan Y Tang WHW Increased need for right ventricular support in patients with chemotherapy-induced cardiomyopathy undergoing mechanical circulatory support: outcomes from the INTERMACS Registry (Interagency Registry for Mechanically Assisted Circulatory Support). *J Am Coll Cardiol.* (2014) 63:240–8. 10.1016/j.jacc.2013.09.040 24161324

[74] Kittipibul V Blumer V Hernandez GA Fudim M Flowers R Chaparro S Pre-operative atrial fibrillation and early right ventricular failure after left ventricular assist device implantation: a systematic review and meta-analysis. *Am Heart J.* (2021) 239:120–8. 10.1016/j.ahj.2021.05.009 34038705

[75] Potapov EV Schoenrath F Falk V. Clinical signs of right ventricular failure following implantation of a left ventricular assist device. *Eur J Heart Fail.* (2020) 22:383–4. 10.1002/ejhf.1657 31777139

[76] Marco G Robert N. Pulmonary hypertension in heart failure. *J Am Coll Cardiol.* (2017) 69:1718–34. 10.1016/j.jacc.2017.01.051 28359519

[77] Krishnamurthy Y Cooper LB Parikh KS Felker GM Milano CA Rogers JG Pulmonary hypertension in the era of mechanical circulatory support. *ASAIO J.* (2016) 62:505–12. 10.1097/MAT.0000000000000408 27442856 PMC5001897

[78] Hayek S Sims DB Markham DW Butler J Kalogeropoulos AP. Assessment of right ventricular function in left ventricular assist device candidates. *Circ Cardiovasc Imaging.* (2014) 7:379–89. 10.1161/CIRCIMAGING.113.001127 24642920 PMC3961845

[79] Chriqui L-E Monney P Kirsch M Tozzi P. Prediction of right ventricular failure after left ventricular assist device implantation in patients with heart failure: a meta-analysis comparing echocardiographic parameters. *Interact Cardiovasc Thorac Surg.* (2021) 33:784–92. 10.1093/icvts/ivab177 34368839 PMC8691721

[80] Raymer DS Moreno JD Sintek MA Nassif ME Sparrow CT Adamo L The combination of tricuspid annular plane systolic excursion and HeartMate risk score predicts right ventricular failure after left ventricular assist device implantation. *ASAIO J.* (2019) 65:247–51. 10.1097/MAT.0000000000000808 29734261 PMC6203672

[81] Patil NP Mohite PN Sabashnikov A Dhar D Weymann A Zeriouh M Preoperative predictors and outcomes of right ventricular assist device implantation after continuous-flow left ventricular assist device implantation. *J Thorac Cardiovasc Surg.* (2015) 150:1651–8. 10.1016/j.jtcvs.2015.07.090 26318358

[82] Dandel M Potapov E Krabatsch T Stepanenko A Löw A Vierecke J Load dependency of right ventricular performance is a major factor to be considered in decision making before ventricular assist device implantation. *Circulation.* (2013) 128(11 Suppl. 1):S14–23. 10.1161/CIRCULATIONAHA.112.000335 24030398

[83] Kato TS Jiang J Schulze PC Jorde U Uriel N Kitada S Serial echocardiography using tissue Doppler and Speckle tracking imaging to monitor right ventricular failure before and after left ventricular assist device surgery. *JACC Heart Fail.* (2013) 1:216–22. 10.1016/j.jchf.2013.02.005 24621873 PMC3997790

[84] Raina A Seetha Rammohan HR Gertz ZM Rame JE Woo YJ Kirkpatrick JN. Postoperative right ventricular failure after left ventricular assist device placement is predicted by preoperative echocardiographic structural, hemodynamic, and functional parameters. *J Card Fail.* (2013) 19:16–24. 10.1016/j.cardfail.2012.11.001 23273590

[85] Kukucka M Stepanenko A Potapov E Krabatsch T Redlin M Mladenow A Right-to-left ventricular end-diastolic diameter ratio and prediction of right ventricular failure with continuous-flow left ventricular assist devices. *J Heart Lung Transplant.* (2011) 30:64–9. 10.1016/j.healun.2010.09.006 21036066

[86] Vivo RP Cordero-Reyes AM Qamar U Garikipati S Trevino AR Aldeiri M Increased right-to-left ventricle diameter ratio is a strong predictor of right ventricular failure after left ventricular assist device. *J Heart Lung Transplant.* (2013) 32:792–9. 10.1016/j.healun.2013.05.016 23856216

[87] Gulati AA Freed KA Florido R Gilotra NA Sharma K Tedford RJ Right ventricular spherical dilatation combined with pulmonary artery compliance predicts severe-acute right heart failure after LVAD implantation. *J Card Fail.* (2019) 25(8 Suppl.):S169. 10.1016/j.cardfail.2019.07.481

[88] Cameli M Loiacono F Sparla S Solari M Iardino E Mandoli GE Systematic left ventricular assist device implant eligibility with non-invasive assessment: the SIENA protocol. *J Cardiovasc Ultrasound.* (2017) 25:39–46. 10.4250/jcu.2017.25.2.39 28770031 PMC5526884

[89] Barssoum K Altibi AM Rai D Kharsa A Kumar A Chowdhury M Assessment of right ventricular function following left ventricular assist device (LVAD) implantation-the role of speckle-tracking echocardiography: a meta-analysis. *Echocardiography.* (2020) 37:2048–60. 10.1111/echo.14884 33084128

[90] Gumus F Durdu MS Cakici M Kurklu TST Inan MB Dincer I Right ventricular free wall longitudinal strain and stroke work index for predicting right heart failure after left ventricular assist device therapy. *Interact Cardiovasc Thorac Surg.* (2019) 28:674–82. 10.1093/icvts/ivy328 30561630

[91] Isaza N Gonzalez M Saijo Y Vega Brizneda M Estep J Starling RC Incremental value of global longitudinal strain to michigan risk score and pulmonary artery pulsatility index in predicting right ventricular failure following left ventricular assist devices. *Heart Lung Circ.* (2022) 31:1110–8. 10.1016/j.hlc.2022.03.012 35491337

[92] Cameli M Lisi M Righini FM Focardi M Lunghetti S Bernazzali S Speckle tracking echocardiography as a new technique to evaluate right ventricular function in patients with left ventricular assist device therapy. *J Heart Lung Transplant.* (2013) 32:424–30. 10.1016/j.healun.2012.12.010 23498163

[93] Grant ADM Smedira NG Starling RC Marwick TH. Independent and incremental role of quantitative right ventricular evaluation for the prediction of right ventricular failure after left ventricular assist device implantation. *J Am Coll Cardiol.* (2012) 60:521–8. 10.1016/j.jacc.2012.02.073 22858287

[94] Magunia H Dietrich C Langer HF Schibilsky D Schlensak C Rosenberger P 3D echocardiography derived right ventricular function is associated with right ventricular failure and mid-term survival after left ventricular assist device implantation. *Int J Cardiol.* (2018) 272:348–55. 10.1016/j.ijcard.2018.06.026 29903518

[95] Kiernan MS French AL DeNofrio D Parmar YJ Pham DT Kapur NK Preoperative three-dimensional echocardiography to assess risk of right ventricular failure after left ventricular assist device surgery. *J Card Fail.* (2015) 21:189–97. 10.1016/j.cardfail.2014.12.009 25535957

[96] Bowen DJ Yalcin YC Strachinaru M McGhie JS van den Bosch AE Soliman OI Right ventricular functional assessment by 2D multi-plane echocardiography prior to left ventricular assist device implantation. *Echocardiography.* (2022) 39:7–19. 10.1111/echo.15191 34877695 PMC9300057

[97] Shah H Maharaj V Kenny B Kalra R El Rafei A Duval S Non-invasive TAPSE/PASP ratio is not predictive of early right ventricular failure post LVAD implantation. *J Heart Lung Transplant.* (2020) 39:S427–8. 10.1016/j.healun.2020.01.218

[98] Dandel M Javier MFDM Del Javier Delmo EM Hetzer R. Accurate assessment of right heart function before and after long-term left ventricular assist device implantation. *Expert Rev Cardiovasc Ther.* (2020) 18:289–308. 10.1080/14779072.2020.1761790 32437212

[99] Charisopoulou D Banner NR Demetrescu C Simon AR Rahman Haley S. Right atrial and ventricular echocardiographic strain analysis predicts requirement for right ventricular support after left ventricular assist device implantation. *Eur Heart J Cardiovasc Imaging.* (2019) 20:199–208. 10.1093/ehjci/jey065 29668926

[100] Alnsasra H Asleh R Schettle SD Pereira NL Frantz RP Edwards BS Diastolic pulmonary gradient as a predictor of right ventricular failure after left ventricular assist device implantation. *J Am Heart Assoc.* (2019) 8:e012073. 10.1161/JAHA.119.012073 31411097 PMC6759881

[101] Tedford RJ Hassoun PM Mathai SC Girgis RE Russell SD Thiemann DR Pulmonary capillary wedge pressure augments right ventricular pulsatile loading. *Circulation.* (2012) 125:289–97. 10.1161/CIRCULATIONAHA.111.051540 22131357 PMC3264431

[102] Grandin EW Zamani P Mazurek JA Troutman GS Birati EY Vorovich E Right ventricular response to pulsatile load is associated with early right heart failure and mortality after left ventricular assist device. *J Heart Lung Transplant.* (2017) 36:97–105. 10.1016/j.healun.2016.06.015 27469015

[103] Tampakakis E Shah SJ Borlaug BA Leary PJ Patel HH Miller WL Pulmonary effective arterial elastance as a measure of right ventricular afterload and its prognostic value in pulmonary hypertension due to left heart disease. *Circ Heart Fail.* (2018) 11:e004436. 10.1161/CIRCHEARTFAILURE.117.004436 29643065 PMC5901761

[104] Essandoh M Kumar N Hussain N Dalia AA Wang D Al-Qudsi O Pulmonary artery pulsatility index as a predictor of right ventricular failure in left ventricular assist device recipients: a systematic review. *J Heart Lung Transplant.* (2022) 41:1114–23. 10.1016/j.healun.2022.04.007 35644726

[105] Beach LY Hiesinger W Wheeler MT. Abstract 15452: low Dp/dtMax, when indexed to central venous pressure, is associated with severe right ventricular failure following left ventricular assist device implantation. *Circulation.* (2020) 142(Suppl. 3):A15452. 10.1161/circ.142.suppl_3.15452

[106] Li DL Agrawal V Hernandez G Sandhaus EM Womack S Kim E Abstract 15339: prediction of severe right ventricular failure after left ventricle assist device using echocardiographic right ventricular Dp/dt. *Circulation.* (2020) 142(Suppl. 3):A15339. 10.1161/circ.142.suppl_3.15339

[107] Samura T Yoshioka D Asanoi H Toda K Miyagawa S Yoshikawa Y Right atrial pressure waveform predicts right ventricular failure after left ventricular assist device implantation. *Ann Thorac Surg.* (2019) 108:1361–8. 10.1016/j.athoracsur.2019.04.050 31175868

[108] Tecson KM Lima B Lee AY Raza FS Ching G Lee C-H Determinants and outcomes of vasoplegia following left ventricular assist device implantation. *J Am Heart Assoc.* (2018) 7:e008377. 10.1161/JAHA.117.008377 29773577 PMC6015358

[109] Mohamedali B Doukky R Karavalos K Avery E Bhat G. Mean arterial pressure to central venous pressure ratio: a novel marker for right ventricular failure after left ventricular assist device placement. *J Card Fail.* (2017) 23:446–52. 10.1016/j.cardfail.2017.03.009 28365215

[110] Morine KJ Kiernan MS Pham DT Paruchuri V Denofrio D Kapur NK. Pulmonary artery pulsatility index is associated with right ventricular failure after left ventricular assist device surgery. *J Card Fail.* (2016) 22:110–6. 10.1016/j.cardfail.2015.10.019 26564619

[111] Kang G Ha R Banerjee D. Pulmonary artery pulsatility index predicts right ventricular failure after left ventricular assist device implantation. *J Heart Lung Transplant.* (2016) 35:67–73. 10.1016/j.healun.2015.06.009 26212656

[112] Aslam MI Jani V Lin BL Dunkerly-Eyring B Livingston CE Ramachandran A Pulmonary artery pulsatility index predicts right ventricular myofilament dysfunction in advanced human heart failure. *Eur J Heart Fail.* (2021) 23:339–41. 10.1002/ejhf.2084 33347674 PMC8574988

[113] Imamura T Kinugawa K Kinoshita O Nawata K Ono M. High pulmonary vascular resistance in addition to low right ventricular stroke work index effectively predicts biventricular assist device requirement. *J Artif Organs.* (2016) 19:44–53. 10.1007/s10047-015-0867-4 26395777

[114] Wright SP Groves L Vishram-Nielsen JKK Karvasarski E Valle FH Alba AC Elevated pulmonary arterial elastance and right ventricular uncoupling are associated with greater mortality in advanced heart failure. *J Heart Lung Transplant.* (2020) 39:657–65. 10.1016/j.healun.2020.02.013 32184043

[115] Gonzalez MH Wang Q Yaranov DM Albert C Wolski K Wagener J Dynamic assessment of pulmonary artery pulsatility index provides incremental risk assessment for early right ventricular failure after left ventricular assist device. *J Card Fail.* (2021) 27:777–85. 10.1016/j.cardfail.2021.02.012 33640481 PMC10257976

[116] Gulati G Sutaria N Vest AR Denofrio DD KawaborI M Couper G Timing and trends of right atrial pressure and risk of right heart failure after left ventricular assist device implantation. *J Card Fail.* (2020) 26:394–401. 10.1016/j.cardfail.2020.01.013 31981695 PMC11081028

[117] Kawabori M Nordan T Kapur NK Couper GS. Protect right: right ventricular failure prevention strategy for left ventricular assist device implantation. *Eur J Cardiothorac Surg.* (2021) 59:1128–30. 10.1093/ejcts/ezaa400 33236106

[118] Cacioli G Polizzi V Ciabatti M Cristiano E Pergolini A Distefano G Prediction of right ventricular failure after left ventricular assist device implantation: role of vasodilator challenge. *Eur Heart J Acute Cardiovasc Care.* (2022) 11:629–39. 10.1093/ehjacc/zuac085 35866303

[119] Read JM Azih NI Peters CJ Gurtu V Vishram-Nielsen JK Wright SP Hemodynamic reserve predicts early right heart failure after LVAD implantation. *J Heart Lung Transplant.* (2022). 10.1016/j.healun.2022.07.003 [Epub ahead of print]. 35934606 PMC10729844

[120] Yourshaw JP Mishra P Armstrong MC Ramu B Craig ML Van Bakel AB Effects of percutaneous LVAD support on right ventricular load and adaptation. *J Cardiovasc Transl Res.* (2019) 12:142–9. 10.1007/s12265-018-9806-0 29713934

[121] Hsi B Joseph D Trachtenberg B Bhimaraj A Suarez EE Xu J Degree of change in right ventricular adaptation measures during axillary impella support informs risk stratification for early, severe right heart failure following durable LVAD implantation. *J Heart Lung Transplant.* (2022) 41:279–82. 10.1016/j.healun.2021.11.007 34998630

[122] Loforte A Montalto A Musumeci F Amarelli C Mariani C Polizzi V Calculation of the ALMA risk of right ventricular failure after left ventricular assist device implantation. *ASAIO J.* (2018) 64:e140–7. 10.1097/MAT.0000000000000800 29746312

[123] Matthews JC Koelling TM Pagani FD Aaronson KD. The right ventricular failure risk score a pre-operative tool for assessing the risk of right ventricular failure in left ventricular assist device candidates. *J Am Coll Cardiol.* (2008) 51:2163–72. 10.1016/j.jacc.2008.03.009 18510965 PMC2842901

[124] Soliman OII Akin S Muslem R Boersma E Manintveld OC Krabatsch T Derivation and validation of a novel right-sided heart failure model after implantation of continuous flow left ventricular assist devices: the EUROMACS (European Registry for Patients with Mechanical Circulatory Support) right-sided heart failure risk S. *Circulation.* (2018) 137:891–906. 10.1161/CIRCULATIONAHA.117.030543 28847897

[125] Loghmanpour NA Kormos RL Kanwar MK Teuteberg JJ Murali S Antaki JF. A Bayesian model to predict right ventricular failure following left ventricular assist device therapy. *JACC Heart Fail.* (2016) 4:711–21. 10.1016/j.jchf.2016.04.004 27289403 PMC5010475

[126] Houston BA Kalathiya RJ Hsu S Loungani R Davis ME Coffin ST Right ventricular afterload sensitivity dramatically increases after left ventricular assist device implantation: a multi-center hemodynamic analysis. *J Heart Lung Transplant.* (2016) 35:868–76. 10.1016/j.healun.2016.01.1225 27041496 PMC4956565

[127] Unsworth B Casula RP Kyriacou AA Yadav H Chukwuemeka A Cherian A The right ventricular annular velocity reduction caused by coronary artery bypass graft surgery occurs at the moment of pericardial incision. *Am Heart J.* (2010) 159:314–22. 10.1016/j.ahj.2009.11.013 20152232 PMC2822903

[128] Borlaug BA Schaff HV Pochettino A Pedrotty DM Asirvatham SJ Abel MD Pericardiotomy enhances left ventricular diastolic reserve with volume loading in humans. *Circulation.* (2018) 138:2295–7. 10.1161/CIRCULATIONAHA.118.036006 30571519 PMC6363353

[129] Lehmann KG Lee FA McKenzie WB Barash PG Prokop EK Durkin MA Onset of altered interventricular septal motion during cardiac surgery. Assessment by continuous intraoperative transesophageal echocardiography. *Circulation.* (1990) 82:1325–34. 10.1161/01.CIR.82.4.1325 2401066

[130] Salas De Armas IA Patel JA Akay MH Patel MK Rajagopal K Karabulut MN Off-pump continuous-flow left ventricular assist device implantation. *Texas Heart Inst J.* (2021) 48:e197033. 10.14503/THIJ-19-7033 33946106 PMC8108714

[131] McGee EJ Danter M Strueber M Mahr C Mokadam NA Wieselthaler G Evaluation of a lateral thoracotomy implant approach for a centrifugal-flow left ventricular assist device: the LATERAL clinical trial. *J Heart Lung Transplant.* (2019) 38:344–51. 10.1016/j.healun.2019.02.002 30945636

[132] Schmitto JD Krabatsch T Damme L Netuka I. Less invasive HeartMate 3 left ventricular assist device implantation. *J Thorac Dis.* (2018) 10(Suppl. 15):S1692–5. 10.21037/jtd.2018.01.26 30034840 PMC6035962

[133] Gosev I Wood K Ayers B Barrus B Knight P Alexis JD Implantation of a fully magnetically levitated left ventricular assist device using a sternal-sparing surgical technique. *J Heart Lung Transplant.* (2020) 39:37–44. 10.1016/j.healun.2019.09.012 31636043

[134] Sileshi B Haglund NA Davis ME Tricarico NM Stulak JM Khalpey Z In-hospital outcomes of a minimally invasive off-pump left thoracotomy approach using a centrifugal continuous-flow left ventricular assist device. *J Heart Lung Transplant.* (2015) 34:107–12. 10.1016/j.healun.2014.09.023 25447579

[135] Attisani M Pocar M Lodo V Barbero C Marchetto G Centofanti P Off-pump left ventricular assist device implantation via bilateral mini-thoracotomy in cardiac reoperations: the extrapericardial subxiphoid route. *Ann Cardiothorac Surg.* (2021) 10:298–300. 10.21037/acs-2020-cfmcs-25 33842229 PMC8033262

[136] Lampert BC Teuteberg JJ Cowger J Mokadam NA Cantor RS Benza RL Impact of thoracotomy approach on right ventricular failure and length of stay in left ventricular assist device implants: an intermacs registry analysis. *J Heart Lung Transplant.* (2021) 40:981–9. 10.1016/j.healun.2021.05.022 34229917

[137] Zhang B Guo S Fu Z Liu Z. Minimally invasive versus conventional continuous-flow left ventricular assist device implantation for heart failure: a meta-analysis. *Heart Fail Rev.* (2021) 27:1053–61. 10.1007/s10741-021-10102-z 33811570

[138] Mariani S Li T Boethig D Napp LC Chatterjee A Homann K Lateral thoracotomy for ventricular assist device implantation: a meta-analysis of literature. *ASAIO J.* (2021) 67:845–55. 10.1097/MAT.0000000000001359 33620165

[139] Shore S Hanff TC Mazurek JA Seigerman M Zhang R Grandin EW The effect of transfusion of blood products on ventricular assist device support outcomes. *ESC Heart Fail.* (2020) 7:3573–81. 10.1002/ehf2.12780 33263224 PMC7754735

[140] Nelson JA Diaz Soto JC Warner MA Stulak JM Schulte PJ Weister TJ Use of plasma late on cardiopulmonary bypass in patients undergoing left ventricular assist device implantation. *Artif Organs.* (2022) 46:491–500. 10.1111/aor.14052 34403155 PMC8850532

[141] Vasques F Spiezia L Manfrini A Tarzia V Fichera D Simioni P Thromboelastometry guided fibrinogen replacement therapy in cardiac surgery: a retrospective observational study. *J Anesth.* (2017) 31:286–90. 10.1007/s00540-016-2271-5 27757554

[142] Opfermann P Felli A Schlömmer C Dworschak M Bevilacqua M Mouhieddine M A prospective observational study on multiplate(§)-, ROTEM(§)- and thrombin generation examinations before and early after implantation of a left ventricular assist device (LVAD). *Front Med.* (2022) 9:760816. 10.3389/fmed.2022.760816 35280873 PMC8914262

[143] Pagano D Milojevic M Meesters MI Benedetto U Bolliger D von Heymann C 2017 EACTS/EACTA guidelines on patient blood management for adult cardiac surgery. *Eur J Cardiothorac Surg.* (2018) 53:79–111. 10.1093/ejcts/ezx325 29029100

[144] Stulak JM Romans T Cowger J Romano MA Haft JW Aaronson KD Delayed sternal closure does not increase late infection risk in patients undergoing left ventricular assist device implantation. *J Heart Lung Transplant.* (2012) 31:1115–9. 10.1016/j.healun.2012.08.015 22975102

[145] Quader M LaPar DJ Wolfe L Ailawadi G Rich J Speir A Delayed sternal closure after continuous flow left ventricle assist device implantation: analysis of risk factors and impact on outcomes and costs. *ASAIO J.* (2016) 62:432–7. 10.1097/MAT.0000000000000384 27164037

[146] Yanagida R Rajagopalan N Davenport DL Tribble TA Bradley MA Hoopes CW. Delayed sternal closure does not reduce complications associated with coagulopathy and right ventricular failure after left ventricular assist device implantation. *J Artif Organs.* (2018) 21:46–51. 10.1007/s10047-017-0996-z 28948385 PMC5816763

[147] Lee M-TG Lee C-C Wang H-M Chou T-H Wu M-C Hsueh K-L Hypothermia increases tissue plasminogen activator expression and decreases post-operative intra-abdominal adhesion. *PLoS One.* (2016) 11:e0160627. 10.1371/journal.pone.0160627 27583464 PMC5008742

[148] Mullan C Caraballo C Ravindra NG Miller PE Mori M McCullough M Clinical impact of concomitant tricuspid valve procedures during left ventricular assist device implantation. *J Heart Lung Transplant.* (2020) 39:926–33. 10.1016/j.healun.2020.05.007 32593561

[149] Song HK Gelow JM Mudd J Chien C Tibayan FA Hollifield K Limited utility of tricuspid valve repair at the time of left ventricular assist device implantation. *Ann Thorac Surg.* (2016) 101:2168–74. 10.1016/j.athoracsur.2016.03.040 27139368 PMC4877201

[150] Veen KM Mokhles MM Soliman O de By TMMH Mohacsi P Schoenrath F Clinical impact and “natural” course of uncorrected tricuspid regurgitation after implantation of a left ventricular assist device: an analysis of the European Registry for Patients with Mechanical Circulatory Support (EUROMACS). *Eur J Cardiothorac Surg.* (2021) 59:207–16. 10.1093/ejcts/ezaa294 33038216 PMC7781523

[151] Mulzer J Krastev H Hoermandinger C Meyer A Haese T Stein J Development of tricuspid regurgitation and right ventricular performance after implantation of centrifugal left ventricular assist devices. *Ann Cardiothorac Surg.* (2021) 10:364–74. 10.21037/acs-2020-cfmcs-fs-0215 34159117 PMC8185391

[152] Barac YD Nicoara A Bishawi M Schroder JN Daneshmand MA Hashmi NK Durability and efficacy of tricuspid valve repair in patients undergoing left ventricular assist device implantation. *JACC Heart Fail.* (2020) 8:141–50. 10.1016/j.jchf.2019.08.016 31838034 PMC6995411

[153] Nakanishi K Homma S Han J Takayama H Colombo PC Yuzefpolskaya M Prevalence, predictors, and prognostic value of residual tricuspid regurgitation in patients with left ventricular assist device. *J Am Heart Assoc.* (2018) 7:e008813. 10.1161/JAHA.118.008813 29937432 PMC6064878

[154] Robertson JO Grau-Sepulveda MV Okada S O’Brien SM Matthew Brennan J Shah AS Concomitant tricuspid valve surgery during implantation of continuous-flow left ventricular assist devices: a Society of Thoracic Surgeons database analysis. *J Heart Lung Transplant.* (2014) 33:609–17. 10.1016/j.healun.2014.01.861 24661682

[155] Nakanishi K Homma S Han J Takayama H Colombo PC Yuzefpolskaya M Usefulness of tricuspid annular diameter to predict late right sided heart failure in patients with left ventricular assist device. *Am J Cardiol.* (2018) 122:115–20. 10.1016/j.amjcard.2018.03.010 29673504

[156] Goldraich L Kawajiri H Foroutan F Braga J Billia P Misurka J Tricuspid valve annular dilation as a predictor of right ventricular failure after implantation of a left ventricular assist device. *J Card Surg.* (2016) 31:110–6. 10.1111/jocs.12685 26748904

[157] Anwer LA Tchantchaleishvili V Poddi S Daly RC Joyce LD Kushwaha SS Atrial fibrillation should guide prophylactic tricuspid procedures during left ventricular assist device implantation. *ASAIO J.* (2018) 64:586–93. 10.1097/MAT.0000000000000698 29088022

[158] Hayashi H Naka Y Sanchez J Takayama H Kurlansky P Ning Y Influence of atrial fibrillation on functional tricuspid regurgitation in patients with HeartMate 3. *J Am Heart Assoc.* (2021) 10:e018334. 10.1161/JAHA.120.018334 33412902 PMC7955423

[159] Itzhaki Ben Zadok O Ben-Avraham B Barac YD Hammer Y Rubachevski V Shaul A Natural history and prognosis of patients with unrepaired tricuspid regurgitation undergoing implantation of left ventricular assist device. *ASAIO J.* (2022) 68:508–15. 10.1097/MAT.0000000000001521 34261877

[160] Addetia K Harb SC Hahn RT Kapadia S Lang RM. Cardiac implantable electronic device lead-induced tricuspid regurgitation. *JACC Cardiovasc Imaging.* (2019) 12:622–36. 10.1016/j.jcmg.2018.09.028 30947905

[161] Robertson JO Naftel DC Myers SL Tedford RJ Joseph SM Kirklin JK Concomitant mitral valve procedures in patients undergoing implantation of continuous-flow left ventricular assist devices: an INTERMACS database analysis. *J Heart Lung Transplant.* (2018) 37:79–88. 10.1016/j.healun.2017.09.016 29150326

[162] Cruz Rodriguez JB Chatterjee A Pamboukian SV Tallaj JA Joly J Lenneman A Persistent mitral regurgitation after left ventricular assist device: a clinical conundrum. *ESC Heart Fail.* (2021) 8:1039–46. 10.1002/ehf2.12919 33471962 PMC8006607

[163] Kassis H Cherukuri K Agarwal R Kanwar M Elapavaluru S Sokos GG Significance of residual mitral regurgitation after continuous flow left ventricular assist device implantation. *JACC Heart Fail.* (2017) 5:81–8. 10.1016/j.jchf.2016.09.014 28017353

[164] Jain R Truby LK Topkara VK. Residual mitral regurgitation in patients with left ventricular assist device support – An INTERMACS analysis. *J Heart Lung Transplant.* (2022). 10.1016/j.healun.2022.03.002 [Epub ahead of print]. 35379546

[165] Gulati G Ruthazer R Denofrio D Vest AR Kent D Kiernan MS. Understanding longitudinal changes in pulmonary vascular resistance after left ventricular assist device implantation. *J Card Fail.* (2021) 27:552–9. 10.1016/j.cardfail.2021.01.004 33450411 PMC8107116

[166] Tang PC Haft JW Romano MA Bitar A Hasan R Palardy M Right ventricular function and residual mitral regurgitation after left ventricular assist device implantation determines the incidence of right heart failure. *J Thorac Cardiovasc Surg.* (2020) 159:897–905.e4. 10.1016/j.jtcvs.2019.03.089 31101350

[167] Fox H Gyoten T Rojas SV Deutsch M-A Schramm R Rudolph V Safety, mortality, and hemodynamic impact of patients with MitraClip undergoing left ventricular assist device implantation. *J Cardiovasc Transl Res.* (2022) 15:676–86. 10.1007/s12265-021-10178-w 34713397 PMC9213377

[168] Pawale A Itagaki S Parikh A Pinney SP Adams DH Anyanwu AC. Mitral valve repair for severe mitral valve regurgitation during left ventricular assist device implantation. *J Thorac Cardiovasc Surg.* (2019) 157:1841–8.e1. 10.1016/j.jtcvs.2018.12.071 31288361

[169] Sandoval E Singh SK Carillo JA Baldwin ACW Ono M Anand J Impact of concomitant mitral valve repair for severe mitral regurgitation at the time of continuous-flow left ventricular assist device insertion. *Interact Cardiovasc Thorac Surg.* (2017) 25:620–3. 10.1093/icvts/ivx223 28962504

[170] Kanwar MK Rajagopal K Itoh A Silvestry SC Uriel N Cleveland JCJ Impact of left ventricular assist device implantation on mitral regurgitation: an analysis from the MOMENTUM 3 trial. *J Heart Lung Transplant.* (2020) 39:529–37. 10.1016/j.healun.2020.03.003 32279919

[171] Imamura T Adatya S Chung B Nguyen A Rodgers D Sayer G Cannula and pump positions are associated with left ventricular unloading and clinical outcome in patients with HeartWare left ventricular assist device. *J Card Fail.* (2018) 24:159–66. 10.1016/j.cardfail.2017.09.013 28982636 PMC5856593

[172] Imamura T Nguyen A Chung B Rodgers D Sarswat N Kim G Association of inflow cannula position with left ventricular unloading and clinical outcomes in patients with HeartMate II left ventricular assist device. *ASAIO J.* (2019) 65:331–5. 10.1097/MAT.0000000000000823 29933250 PMC6342671

[173] Imamura T Narang N Nitta D Fujino T Nguyen A Kim G Optimal cannula positioning of HeartMate 3 left ventricular assist device. *Artif Organs.* (2020) 44:e509–19. 10.1111/aor.13755 32557769

[174] Pasrija C Sawan MA Sorensen E Gammie JS Madathil R Tran D Inflow cannula position influences improvement in mitral regurgitation after ventricular assist device implantation. *ASAIO J.* (2021) 67:423–9. 10.1097/MAT.0000000000001248 33769997

[175] Sayer G Sarswat N Kim GH Adatya S Medvedofsky D Rodgers D The hemodynamic effects of aortic insufficiency in patients supported with continuous-flow left ventricular assist devices. *J Card Fail.* (2017) 23:545–51. 10.1016/j.cardfail.2017.04.012 28435003

[176] Truby LK Garan AR Givens RC Wayda B Takeda K Yuzefpolskaya M Aortic insufficiency during contemporary left ventricular assist device support: analysis of the INTERMACS registry. *JACC Heart Fail.* (2018) 6:951–60. 10.1016/j.jchf.2018.07.012 30384913 PMC6217859

[177] Imamura T Narang N Kim G Nitta D Fujino T Nguyen A Aortic insufficiency during HeartMate 3 left ventricular assist device support. *J Card Fail.* (2020) 26:863–9. 10.1016/j.cardfail.2020.05.013 32473380

[178] Jorde UP Uriel N Nahumi N Bejar D Gonzalez-Costello J Thomas SS Prevalence, significance, and management of aortic insufficiency in continuous flow left ventricular assist device recipients. *Circ Heart Fail.* (2014) 7:310–9. 10.1161/CIRCHEARTFAILURE.113.000878 24415682

[179] Tanaka Y Nakajima T Fischer I Wan F Kotkar K Moon MR The impact of uncorrected mild aortic insufficiency at the time of left ventricular assist device implantation. *J Thorac Cardiovasc Surg.* (2020) 160:1490–500.e3. 10.1016/j.jtcvs.2020.02.144 32998831

[180] Fukuhara S Ikegami H Polanco AR Song JJ Han J Takeda K Concomitant repair for mild aortic insufficiency and continuous-flow left ventricular assist devices. *Eur J Cardiothorac Surg.* (2017) 52:1062–8. 10.1093/ejcts/ezx150 28535190

[181] McKellar SH Deo S Daly RC Durham LA III Joyce LD Stulak JM Durability of central aortic valve closure in patients with continuous flow left ventricular assist devices. *J Thorac Cardiovasc Surg.* (2014) 147:344–8. 10.1016/j.jtcvs.2012.09.098 23246052

[182] Robertson JO Naftel DC Myers SL Prasad S Mertz GD Itoh A Concomitant aortic valve procedures in patients undergoing implantation of continuous-flow left ventricular assist devices: an INTERMACS database analysis. *J Heart Lung Transplant.* (2015) 34:797–805. 10.1016/j.healun.2014.11.008 25511747 PMC4433438

[183] Tang PC Sarsour N Haft JW Romano MA Konerman M Colvin M Aortic valve repair versus replacement associated with durable left ventricular assist devices. *Ann Thorac Surg.* (2020) 110:1259–64. 10.1016/j.athoracsur.2020.01.015 32105716

[184] Fine NM Park SJ Stulak JM Topilsky Y Daly RC Joyce LD Proximal thoracic aorta dimensions after continuous-flow left ventricular assist device implantation: longitudinal changes and relation to aortic valve insufficiency. *J Heart Lung Transplant.* (2016) 35:423–32. 10.1016/j.healun.2015.10.029 26632029

[185] Yoshida S Toda K Miyagawa S Yoshikawa Y Hata H Yoshioka D Impact of turbulent blood flow in the aortic root on de novo aortic insufficiency during continuous-flow left ventricular-assist device support. *Artif Organs.* (2020) 44:883–91. 10.1111/aor.13671 32080864

[186] Iizuka K Nishinaka T Akiyama D Sumikura H Mizuno T Tsukiya T The angle of the outflow graft to the aorta can affect recirculation due to aortic insufficiency under left ventricular assist device support. *J Artif Organs.* (2018) 21:399–404. 10.1007/s10047-018-1064-z 30039455

[187] Kasinpila P Kong S Fong R Shad R Kaiser AD Marsden AL Use of patient-specific computational models for optimization of aortic insufficiency after implantation of left ventricular assist device. *J Thorac Cardiovasc Surg.* (2021) 162:1556–63. 10.1016/j.jtcvs.2020.04.164 32653292 PMC7666659

[188] Gasparovic H Kopjar T Saeed D Cikes M Svetina L Petricevic M De novo aortic regurgitation after continuous-flow left ventricular assist device implantation. *Ann Thorac Surg.* (2017) 104:704–11. 10.1016/j.athoracsur.2017.01.114 28483150

[189] Grinstein J Kruse E Sayer G Fedson S Kim GH Sarswat N Novel echocardiographic parameters of aortic insufficiency in continuous-flow left ventricular assist devices and clinical outcome. *J Heart Lung Transplant.* (2016) 35:976–85. 10.1016/j.healun.2016.05.009 27373822 PMC5393266

[190] Grinstein J Kruse E Sayer G Fedson S Kim GH Jorde UP Accurate quantification methods for aortic insufficiency severity in patients with LVAD: role of diastolic flow acceleration and systolic-to-diastolic peak velocity ratio of outflow cannula. *JACC Cardiovasc Imaging.* (2016) 9:641–51. 10.1016/j.jcmg.2015.06.020 26684975

[191] Yehya A Rajagopal V Meduri C Kauten J Brown M Dean L Short-term results with transcatheter aortic valve replacement for treatment of left ventricular assist device patients with symptomatic aortic insufficiency. *J Heart Lung Transplant.* (2019) 38:920–6. 10.1016/j.healun.2019.03.001 30898555

[192] Dhillon AS Jones BM Hodson RW Korngold EC. Transcatheter aortic valve replacement for severe aortic regurgitation in patients with a left ventricular assist device. *J Invasive Cardiol.* (2022) 34:E369–73. 35343915 10.25270/jic/21.00212

[193] Phan K Haswell JM Xu J Assem Y Mick SL Kapadia SR Percutaneous transcatheter interventions for aortic insufficiency in continuous-flow left ventricular assist device patients: a systematic review and meta-analysis. *ASAIO J.* (2017) 63:117–22. 10.1097/MAT.0000000000000447 27676407

[194] Lanmueller P Eulert-Grehn J-J Unbehaun A Klein C Hommel M Kofler M Interventional procedures for left ventricular assist device-associated complications. *ASAIO J.* (2022). 10.1097/MAT.0000000000001674 [Epub ahead of print]. 35184090

[195] Beukian S Lala A Dangas G Tang GHL Anyanwu A Moss N Subacute aortic root and valve thrombosis following transcatheter aortic valve replacement in a left ventricular assist device patient: from one problem to the next. *CASE.* (2021) 5:97–100. 10.1016/j.case.2020.12.005 33912777 PMC8071813

[196] Rao SD Jagasia D Anwaruddin S Birati EY. Transcatheter aortic valve replacement thrombosis in patient supported with durable left ventricular assist device. *Catheter Cardiovasc Interv.* (2020) 96:500–3. 10.1002/ccd.28742 31977150

[197] Ali J Catarino P Abu-Omar Y. Transcatheter aortic valve implantation in patients with a left ventricular assist device: a word of caution. *Eur J Cardiothorac Surg.* (2020) 58:1309–10. 10.1093/ejcts/ezaa237 32766699

[198] Plymen C Pettit SJ Tsui S Lewis C. Right ventricular failure due to late embolic RV infarction during continuous flow LVAD support. *BMJ Case Rep.* (2015) 2015:bcr2015212174. 10.1136/bcr-2015-212174 26677152 PMC4691854

[199] Nakajima S Seguchi O Murata Y Fujita T Hata H Yamane T Left coronary artery occlusion caused by a large thrombus on the left coronary cusp in a patient with a continuous-flow ventricular assist device. *J Artif Organs.* (2014) 17:197–201. 10.1007/s10047-014-0758-0 24509915

[200] Khan F Maltais S Daly R Dunlay S Stulak J. Abstract 13315: impact of coronary artery disease after left ventricular assist device implantation: incidence, manifestations, and interventions. *Circulation.* (2018) 138(Suppl. 1):A13315.

[201] Çelik M Stulak JM Maltais S. The importance of coronary artery disease and special considerations for left ventricular assist device implantation. *Ann Cardiothorac Surg.* (2021) 10:268–70. 10.21037/acs-2020-cfmcs-31 33842221 PMC8033257

[202] Mehta P Imamura T Juricek C Sarswat N Kim G Raikhelkar J Combined left ventricular assist device and coronary artery bypass grafting surgery: should we bypass the bypass? *ASAIO J.* (2020) 66:32–7. 10.1097/MAT.0000000000000956 31294723

[203] Baxter RD Tecson KM Still S Collier JDG Felius J Joseph SM Predictors and impact of right heart failure severity following left ventricular assist device implantation. *J Thorac Dis.* (2019) 11(Suppl. 6):S864–70. 10.21037/jtd.2018.09.155 31183166 PMC6535485

[204] Sparrow CT Nassif ME Raymer DS Novak E LaRue SJ Schilling JD. Pre-operative right ventricular dysfunction is associated with gastrointestinal bleeding in patients supported with continuous-flow left ventricular assist devices. *JACC Heart Fail.* (2015) 3:956–64. 10.1016/j.jchf.2015.09.009 26577618

[205] Joly JM El-Dabh A Kirklin JK Marshell R Smith MG Acharya D High right atrial pressure and low pulse pressure predict gastrointestinal bleeding in patients with left ventricular assist device. *J Card Fail.* (2018) 24:487–93. 10.1016/j.cardfail.2018.03.003 29572191

[206] Carmona A Hoang Minh T Perrier S Schneider C Marguerite S Ajob G Minimally invasive surgery for left ventricular assist device implantation is safe and associated with a decreased risk of right ventricular failure. *J Thorac Dis.* (2020) 12:1496–506. 10.21037/jtd.2020.02.32 32395287 PMC7212123

[207] Choi AY Anand J Bishawi M Halpern SE Contreras FJ Mendiola MA Incidence and diagnostic challenges of bowel ischemia after continuous-flow left ventricular assist device therapy. *ASAIO J.* (2022) 68:676–82. 10.1097/MAT.0000000000001553 34437327 PMC8866539

[208] Reid G Mork C Gahl B Appenzeller-Herzog C von Segesser LK Eckstein F Outcome of right ventricular assist device implantation following left ventricular assist device implantation: systematic review and meta-analysis. *Perfusion.* (2021). 10.1177/02676591211024817 [Epub ahead of print]. 34112048 PMC9619248

[209] Kiernan MS Grandin EW Brinkley MJ Kapur NK Pham DT Ruthazer R Early right ventricular assist device use in patients undergoing continuous-flow left ventricular assist device implantation: incidence and risk factors from the interagency registry for mechanically assisted circulatory support. *Circ Heart Fail.* (2017) 10:e003863. 10.1161/CIRCHEARTFAILURE.117.003863 29021348 PMC5717751

[210] Marzec LN Ambardekar AV. Preoperative evaluation and perioperative management of right ventricular failure after left ventricular assist device implantation. *Semin Cardiothorac Vasc Anesth.* (2013) 17:249–61. 10.1177/1089253213488246 23640105

[211] Klima U Guerrero JL Vlahakes GJ. Contribution of the interventricular septum to maximal right ventricular function. *Eur J Cardiothorac Surg.* (1998) 14:250–5. 10.1016/S1010-7940(98)00179-1 9761433

[212] Gebhardt BR Abdulaziz A Kawabori M Gudejko MD Cobey FC. Intraoperative central venous pressure and diastolic pulmonary artery pressure as a marker of severe right ventricular failure after left ventricular assist device implantation. *J Cardiothorac Vasc Anesthes.* (2020) 34:847–9. 10.1053/j.jvca.2019.08.043 31570242

[213] Gudejko MD Gebhardt BR Zahedi F Jain A Breeze JL Lawrence MR Intraoperative hemodynamic and echocardiographic measurements associated with severe right ventricular failure after left ventricular assist device implantation. *Anesth Analg.* (2019) 128:25–32. 10.1213/ANE.0000000000003538 29878942 PMC7908049

[214] Cordtz J Nilsson JC Hansen PB Sander K Olesen PS Boesgaard S Right ventricular failure after implantation of a continuous-flow left ventricular assist device: early haemodynamic predictors. *Eur J Cardiothorac Surg.* (2014) 45:847–53. 10.1093/ejcts/ezt519 24258201

[215] Balthazar T Adriaenssens T Rega F Vandenbriele C. Pulsus alternans as a sign of right ventricular failure after left ventricular assist device implantation. *J Card Fail.* (2020) 26:1093–5. 10.1016/j.cardfail.2020.09.010 32956812

[216] Alfirevic A Makarova N Kelava M Sale S Soltesz E Duncan AE. Predicting right ventricular failure after LVAD implantation: role of tricuspid valve annulus displacement. *J Cardiothorac Vasc Anesth.* (2020) 34:1204–10. 10.1053/j.jvca.2019.08.045 31558395

[217] Silverton NA Patel R Zimmerman J Ma J Stoddard G Selzman CH Intraoperative transesophageal echocardiography and right ventricular failure after left ventricular assist device implantation. *J Cardiothorac Vasc Anesth.* (2018) 32:2096–103. 10.1053/j.jvca.2018.02.023 29555387

[218] Bahatyrevich N Yang Q Cavarocchi NC Hirose H. Is hemodynamic transesophageal echocardiography needed for patients with left ventricular assist device? *J Thorac Cardiovasc Surg.* (2018) 155:1071–7. 10.1016/j.jtcvs.2017.09.142 29248289

[219] Estrada VHN Franco DLM Moreno AAV Gambasica JAR Nunez CCC. Postoperative Right ventricular failure in cardiac surgery. *Cardiol Res.* (2016) 7:185–95. 10.14740/cr500e 28197291 PMC5295509

[220] Haddad F Doyle R Murphy DJ Hunt SA. Right ventricular function in cardiovascular disease, part II: pathophysiology, clinical importance, and management of right ventricular failure. *Circulation.* (2008) 117:1717–31. 10.1161/CIRCULATIONAHA.107.653584 18378625

[221] Hobbhahn J Habazettl H Conzen P Peter K. [Complications caused by protamine. 1: pharmacology and pathophysiology]. *Anaesthesist.* (1991) 40:365–74. 1928709

[222] Chaney MA Devin Roberts J Wroblewski K Shahul S Gaudet R Jeevanandam V. Protamine administration via the ascending aorta may prevent cardiopulmonary instability. *J Cardiothorac Vasc Anesth.* (2016) 30:647–55. 10.1053/j.jvca.2015.11.014 26948466

[223] Alviar CL Miller PE McAreavey D Katz JN Lee B Moriyama B Positive pressure ventilation in the cardiac intensive care unit. *J Am Coll Cardiol.* (2018) 72:1532–53. 10.1016/j.jacc.2018.06.074 30236315 PMC11032173

[224] Zayat R Menon AK Goetzenich A Schaelte G Autschbach R Stoppe C Benefits of ultra-fast-track anesthesia in left ventricular assist device implantation: a retrospective, propensity score matched cohort study of a four-year single center experience. *J Cardiothorac Surg.* (2017) 12:10. 10.1186/s13019-017-0573-9 28179009 PMC5299681

[225] Ahmad U Khattab MA Schaelte G Goetzenich A Foldenauer AC Moza A Combining minimally invasive surgery with ultra-fast-track anesthesia in HeartMate 3 patients: a pilot study. *Circ Heart Fail.* (2022) 15:e008358. 10.1161/CIRCHEARTFAILURE.121.008358 35249368

[226] Dell’Italia LJ Starling MR Blumhardt R Lasher JC O’Rourke RA. Comparative effects of volume loading, dobutamine, and nitroprusside in patients with predominant right ventricular infarction. *Circulation.* (1985) 72:1327–35. 10.1161/01.CIR.72.6.1327 4064277

[227] Kattan E Castro R Miralles-Aguiar F Hernández G Rola P. The emerging concept of fluid tolerance: a position paper. *J Crit Care.* (2022) 71:154070. 10.1016/j.jcrc.2022.154070 35660844

[228] Jeon Y Ryu JH Lim YJ Kim CS Bahk J-H Yoon SZ Comparative hemodynamic effects of vasopressin and norepinephrine after milrinone-induced hypotension in off-pump coronary artery bypass surgical patients. *Eur J Cardiothorac Surg.* (2006) 29:952–6. 10.1016/j.ejcts.2006.02.032 16675238

[229] Haglund NA Burdorf A Jones T Shostrom V Um J Ryan T Inhaled milrinone after left ventricular assist device implantation. *J Card Fail.* (2015) 21:792–7. 10.1016/j.cardfail.2015.04.011 25937146

[230] Sia YT Gebhard CE Denault AY. Reversal of acute right ventricular failure early post left ventricular assist device placement by intratracheal milrinone administration: case report. *Chest.* (2021) 159:e57–60. 10.1016/j.chest.2020.01.059 33422243

[231] Kocabeyoglu SS Kervan U Sert DE Karahan M Aygun E Beyazal OF Optimization with levosimendan improves outcomes after left ventricular assist device implantation. *Eur J Cardiothorac Surg.* (2020) 57:176–82. 10.1093/ejcts/ezz159 31155645

[232] Mehta RH Leimberger JD van Diepen S Meza J Wang A Jankowich R Levosimendan in patients with left ventricular dysfunction undergoing cardiac surgery. *N Engl J Med.* (2017) 376:2032–42. 10.1056/NEJMoa1616218 28316276

[233] Cholley B Caruba T Grosjean S Amour J Ouattara A Villacorta J Effect of levosimendan on low cardiac output syndrome in patients with low ejection fraction undergoing coronary artery bypass grafting with cardiopulmonary bypass: the LICORN randomized clinical trial. *JAMA.* (2017) 318:548–56. 10.1001/jama.2017.9973 28787507 PMC5817482

[234] Landoni G Lomivorotov VV Alvaro G Lobreglio R Pisano A Guarracino F Levosimendan for hemodynamic support after cardiac surgery. *N Engl J Med.* (2017) 376:2021–31. 10.1056/NEJMoa1616325 28320259

[235] Strong C Raposo L Castro M Madeira S Tralhão A Ventosa A Haemodynamic effects and potential clinical implications of inhaled nitric oxide during right heart catheterization in heart transplant candidates. *ESC Heart Fail.* (2020) 7:673–81. 10.1002/ehf2.12639 32045139 PMC7160504

[236] Griffiths MJD Evans TW. Inhaled nitric oxide therapy in adults. *N Engl J Med.* (2005) 353:2683–95. 10.1056/NEJMra051884 16371634

[237] Potapov E Meyer D Swaminathan M Ramsay M El Banayosy A Diehl C Inhaled nitric oxide after left ventricular assist device implantation: a prospective, randomized, double-blind, multicenter, placebo-controlled trial. *J Heart Lung Transplant.* (2011) 30:870–8. 10.1016/j.healun.2011.03.005 21530317

[238] Argenziano M Choudhri AF Moazami N Rose EA Smith CR Levin HR Randomized, double-blind trial of inhaled nitric oxide in LVAD recipients with pulmonary hypertension. *Ann Thorac Surg.* (1998) 65:340–5. 10.1016/S0003-4975(97)01307-6 9485226

[239] Kukucka M Potapov E Stepanenko A Weller K Mladenow A Kuppe H Acute impact of left ventricular unloading by left ventricular assist device on the right ventricle geometry and function: effect of nitric oxide inhalation. *J Thorac Cardiovasc Surg.* (2011) 141:1009–14. 10.1016/j.jtcvs.2010.08.010 20884019

[240] Chang JC Sawa Y Ohtake S Fukushima N Nishimura M Kagizaki K Hemodynamic effect of inhaled nitric oxide in dilated cardiomyopathy patients on LVAD support. *ASAIO J.* (1997) 43:M418–21. 10.1097/00002480-199709000-00012 9360074

[241] Groves DS Blum FE Huffmyer JL Kennedy JLW Ahmad HB Durieux ME Effects of early inhaled epoprostenol therapy on pulmonary artery pressure and blood loss during LVAD placement. *J Cardiothorac Vasc Anesth.* (2014) 28:652–60. 10.1053/j.jvca.2013.05.028 24103713

[242] Antoniou T Prokakis C Athanasopoulos G Thanopoulos A Rellia P Zarkalis D Inhaled nitric oxide plus iloprost in the setting of post-left assist device right heart dysfunction. *Ann Thorac Surg.* (2012) 94:792–8. 10.1016/j.athoracsur.2012.04.046 22727248

[243] Pagano D Townend JN Horton R Smith C Clutton-Brock T Bonser RS. A comparison of inhaled nitric oxide with intravenous vasodilators in the assessment of pulmonary haemodynamics prior to cardiac transplantation. *Eur J Cardiothorac Surg.* (1996) 10:1120–6. 10.1016/S1010-7940(96)80360-5 10369648

[244] Savaris SL Chang IS Wright SP Mak S. Ramping up the pressure on the right ventricle. *Circ Heart Fail.* (2022) 15:e009671. 10.1161/CIRCHEARTFAILURE.122.009671 35443780

[245] Rosenbaum AN Clavell AL Stulak JM Behfar A. Correction of high afterload improves low cardiac output in patients supported on left ventricular assist device therapy. *ASAIO J.* (2021) 67:32–8. 10.1097/MAT.0000000000001159 32224784

[246] Klodell CTJ Morey TE Lobato EB Aranda JMJ Staples ED Schofield RS Effect of sildenafil on pulmonary artery pressure, systemic pressure, and nitric oxide utilization in patients with left ventricular assist devices. *Ann Thorac Surg.* (2007) 83:68–71; discussion 71. 10.1016/j.athoracsur.2006.08.051 17184632

[247] Tedford RJ Hemnes AR Russell SD Wittstein IS Mahmud M Zaiman AL PDE5A inhibitor treatment of persistent pulmonary hypertension after mechanical circulatory support. *Circ Heart Fail.* (2008) 1:213–9. 10.1161/CIRCHEARTFAILURE.108.796789 19808294 PMC4001820

[248] Critoph C Green G Hayes H Baumwol J Lam K Larbalestier R Clinical outcomes of patients treated with pulmonary vasodilators early and in high dose after left ventricular assist device implantation. *Artif Organs.* (2016) 40:106–14. 10.1111/aor.12502 25994765

[249] Gulati G Grandin EW Kennedy K Cabezas F DeNofrio DD Kociol R Preimplant phosphodiesterase-5 inhibitor use is associated with higher rates of severe early right heart failure after left ventricular assist device implantation. *Circ Heart Fail.* (2019) 12:e005537. 10.1161/CIRCHEARTFAILURE.118.005537 31181953 PMC6624075

[250] Moss N Rakita V Lala A Parikh A Roldan J Mitter SS Hemodynamic response to exercise in patients supported by continuous flow left ventricular assist devices. *JACC Heart Fail.* (2020) 8:291–301. 10.1016/j.jchf.2019.10.013 32035893

[251] Kittipibul V Blumer V Angsubhakorn N Hernandez GA Chaparro S Tedford RJ Phosphodiesterase-5 inhibitors and outcomes during left ventricular assist device support: a systematic review and meta-analysis. *J Card Fail.* (2021) 27:477–85. 10.1016/j.cardfail.2020.12.018 33385522

[252] Parikh U Lamba H Ajmal M Vincent J Walther C Shafii A Predictors of renal replacement therapy in patients with continuous flow left ventricular assist devices. *J Artif Organs.* (2021) 24:207–16. 10.1007/s10047-020-01239-z 33598826

[253] Thongprayoon C Lertjitbanjong P Cheungpasitporn W Hansrivijit P Fülöp T Kovvuru K Incidence and impact of acute kidney injury on patients with implantable left ventricular assist devices: a meta-analysis. *Ren Fail.* (2020) 42:495–512. 10.1080/0886022X.2020.1768116 32434422 PMC7301695

[254] Muslem R Caliskan K Akin S Sharma K Gilotra NA Constantinescu AA Acute kidney injury and 1-year mortality after left ventricular assist device implantation. *J Heart Lung Transplant.* (2018) 37:116–23. 10.1016/j.healun.2017.11.005 29174532

[255] Pasrija C Tran D George P Sorensen E Kaczorowski DJ Ton V-K Left ventricular assist device implantation may be feasible in appropriately selected patients with severe renal insufficiency. *J Thorac Cardiovasc Surg.* (2020) 159:1307–19.e2. 10.1016/j.jtcvs.2019.03.098 31128896

[256] Metkus TS Mullin CJ Grandin EW Rame JE Tampakakis E Hsu S Heart rate dependence of the pulmonary resistance x compliance (RC) time and impact on right ventricular load. *PLoS One.* (2016) 11:e0166463. 10.1371/journal.pone.0166463 27861600 PMC5115737

[257] Deshmukh A Kim G Burke M Anyanwu E Jeevanandam V Uriel N Atrial arrhythmias and electroanatomical remodeling in patients with left ventricular assist devices. *J Am Heart Assoc.* (2017) 6:e005340. 10.1161/JAHA.116.005340 28275069 PMC5524037

[258] Brisco MA Sundareswaran KS Milano CA Feldman D Testani JM Ewald GA Incidence, risk, and consequences of atrial arrhythmias in patients with continuous-flow left ventricular assist devices. *J Card Surg.* (2014) 29:572–80. 10.1111/jocs.12336 24750460

[259] Antonides CFJ Yalcin YC Veen KM Muslem R De By TMMH Bogers AJJC Survival and adverse events in patients with atrial fibrillation at left ventricular assist device implantation: an analysis of the European Registry for Patients with Mechanical Circulatory Support. *Eur J Cardiothorac Surg.* (2022) 61:1164–75. 10.1093/ejcts/ezac023 35076057 PMC9070499

[260] Xia Y Stern D Friedmann P Goldstein D. Preoperative atrial fibrillation may not increase thromboembolic events in left ventricular assist device recipients on midterm follow-up. *J Heart Lung Transplant.* (2016) 35:906–12. 10.1016/j.healun.2016.03.003 27132796

[261] Hottigoudar RU Deam AG Birks EJ McCants KC Slaughter MS Gopinathannair R. Catheter ablation of atrial flutter in patients with left ventricular assist device improves symptoms of right heart failure. *Congest Heart Fail.* (2013) 19:165–71. 10.1111/chf.12034 23910701

[262] Hawkins RB Mehaffey JH Guo A Charles EJ Speir AM Rich JB Postoperative atrial fibrillation is associated with increased morbidity and resource utilization after left ventricular assist device placement. *J Thorac Cardiovasc Surg.* (2018) 156:1543–9.e4. 10.1016/j.jtcvs.2018.03.169 29801690 PMC6156995

[263] Garan AR Levin AP Topkara V Thomas SS Yuzefpolskaya M Colombo PC Early post-operative ventricular arrhythmias in patients with continuous-flow left ventricular assist devices. *J Heart Lung Transplant.* (2015) 34:1611–6. 10.1016/j.healun.2015.05.018 26212658

[264] Galand V Flécher E Auffret V Pichard C Boulé S Vincentelli A Early ventricular arrhythmias after LVAD implantation is the strongest predictor of 30-day post-operative mortality. *JACC Clin Electrophysiol.* (2019) 5:944–54. 10.1016/j.jacep.2019.05.025 31439296

[265] Greet BD Pujara D Burkland D Pollet M Sudhakar D Rojas F Incidence, predictors, and significance of ventricular arrhythmias in patients with continuous-flow left ventricular assist devices: a 15-year institutional experience. *JACC Clin Electrophysiol.* (2018) 4:257–64. 10.1016/j.jacep.2017.11.001 29749947

[266] Gopinathannair R Cornwell WK Dukes JW Ellis CR Hickey KT Joglar JA Device therapy and arrhythmia management in left ventricular assist device recipients: a scientific statement from the American Heart Association. *Circulation.* (2019) 139:e967–89. 10.1161/CIR.0000000000000673 30943783

[267] Martins RP Leclercq C Bourenane H Auffret V Boulé S Loobuyck V Incidence, predictors, and clinical impact of electrical storm in patients with left ventricular assist devices: new insights from the ASSIST-ICD study. *Heart Rhythm.* (2019) 16:1506–12. 10.1016/j.hrthm.2019.06.021 31255846

[268] Rehorn MR Black-Maier E Loungani R Sen S Sun AY Friedman DJ Electrical storm in patients with left ventricular assist devices: risk factors, incidence, and impact on survival. *Heart Rhythm.* (2021) 18:1263–71. 10.1016/j.hrthm.2021.03.047 33839327

[269] Joly JM Acharya D Doppalapudi H Kay GN Lin CP Maddox WR Acute hemodynamic effects of biventricular pacing after left ventricular assist device. *J Card Fail.* (2018) 24:716–8. 10.1016/j.cardfail.2018.09.008 30248397 PMC6640142

[270] Cotarlan V Johnson F Goerbig-Campbell J Light-McGroary K Inampudi C Franzwa J Usefulness of cardiac resynchronization therapy in patients with continuous flow left ventricular assist devices. *Am J Cardiol.* (2019) 123:93–9. 10.1016/j.amjcard.2018.09.022 30539750

[271] Tehrani DM Adatya S Grinstein J Rodgers D Sarswat N Kim GH Impact of cardiac resynchronization therapy on left ventricular unloading in patients with implanted left ventricular assist devices. *ASAIO J.* (2019) 65:117–22. 10.1097/MAT.0000000000000787 29608492 PMC6221999

[272] Tomashitis B Baicu CF Butschek RA Jackson GR Winterfield J Tedford RJ Acute hemodynamic effects of cardiac resynchronization therapy versus alternative pacing strategies in patients with left ventricular assist devices. *J Am Heart Assoc.* (2021) 10:e018127. 10.1161/JAHA.120.018127 33663225 PMC8174219

[273] Chung BB Grinstein JS Imamura T Kruse E Nguyen AB Narang N Biventricular pacing versus right ventricular pacing in patients supported with LVAD. *JACC Clin Electrophysiol.* (2021) 7:1003–9. 10.1016/j.jacep.2021.01.016 34217657

[274] Gopinathannair R Roukoz H Bhan A Ravichandran A Ahmed MM Familtsev D Cardiac resynchronization therapy and clinical outcomes in continuous flow left ventricular assist device recipients. *J Am Heart Assoc.* (2018) 7:e009091. 10.1161/JAHA.118.009091 29907652 PMC6220540

[275] Takeda K Naka Y Yang JA Uriel N Colombo PC Jorde UP Outcome of unplanned right ventricular assist device support for severe right heart failure after implantable left ventricular assist device insertion. *J Heart Lung Transplant.* (2014) 33:141–8. 10.1016/j.healun.2013.06.025 23932442

[276] Fitzpatrick JR III Frederick JR Hiesinger W Hsu VM McCormick RC Kozin ED Early planned institution of biventricular mechanical circulatory support results in improved outcomes compared with delayed conversion of a left ventricular assist device to a biventricular assist device. *J Thorac Cardiovasc Surg.* (2009) 137:971–7. 10.1016/j.jtcvs.2008.09.021 19327526 PMC3232461

[277] Lazar JF Swartz MF Schiralli MP Schneider M Pisula B Hallinan W Survival after left ventricular assist device with and without temporary right ventricular support. *Ann Thorac Surg.* (2013) 96:2155–9. 10.1016/j.athoracsur.2013.07.008 24035303

[278] Beller JP Mehaffey JH Wegermann ZK Grau-Sepulveda M O’Brien SM Brennan JM Strategies for mechanical right ventricular support during left ventricular assist device implant. *Ann Thorac Surg.* (2021) 114:484–1. 10.1016/j.athoracsur.2021.10.032 34843696

[279] Fischer Q Kirsch M. Liberal right ventricular assist device extracorporeal membrane oxygenation support for right ventricular failure after implantable left ventricular assist device placement. *ASAIO J.* (2018) 64:741–7. 10.1097/MAT.0000000000000735 29210774

[280] Riebandt J Haberl T Wiedemann D Moayedifar R Schloeglhofer T Mahr S Extracorporeal membrane oxygenation support for right ventricular failure after left ventricular assist device implantation. *Eur J Cardiothorac Surg.* (2018) 53:590–5. 10.1093/ejcts/ezx349 29045747

[281] Scherer M Sirat AS Moritz A Martens S. Extracorporeal membrane oxygenation as perioperative right ventricular support in patients with biventricular failure undergoing left ventricular assist device implantation. *Eur J Cardiothorac Surg.* (2011) 39:939–44; discussion 944. 10.1016/j.ejcts.2010.09.044 21071240

[282] Coromilas EJ Takeda K Ando M Cevasco M Green P Karmpaliotis D Comparison of percutaneous and surgical right ventricular assist device support after durable left ventricular assist device insertion. *J Card Fail.* (2019) 25:105–13. 10.1016/j.cardfail.2018.12.005 30582967 PMC6377854

[283] Leidenfrost J Prasad S Itoh A Lawrance CP Bell JM Silvestry SC. Right ventricular assist device with membrane oxygenator support for right ventricular failure following implantable left ventricular assist device placement. *Eur J Cardiothorac Surg.* (2016) 49:73–7. 10.1093/ejcts/ezv116 25877948

[284] Kapur NK Esposito ML Bader Y Morine KJ Kiernan MS Pham DT Mechanical circulatory support devices for acute right ventricular failure. *Circulation.* (2017) 136:314–26. 10.1161/CIRCULATIONAHA.116.025290 28716832

[285] Salna M Garan AR Kirtane AJ Karmpaliotis D Green P Takayama H Novel percutaneous dual-lumen cannula-based right ventricular assist device provides effective support for refractory right ventricular failure after left ventricular assist device implantation. *Interact Cardiovasc Thorac Surg.* (2020) 30:499–506. 10.1093/icvts/ivz322 31986207

[286] Joshi Y Bories M-C Aissaoui N Grinda J-M Bel A Latremouille C Percutaneous venopulmonary artery extracorporeal membrane oxygenation for right heart failure after left ventricular assist device insertion. *Interact Cardiovasc Thorac Surg.* (2021) 33:978–85. 10.1093/icvts/ivab197 34313320 PMC8923379

[287] Abdelshafy M Caliskan K Guven G Elkoumy A Elsherbini H Elzomor H Temporary right-ventricular assist devices: a systematic review. *J Clin Med.* (2022) 11:613. 10.3390/jcm11030613 35160064 PMC8837135

[288] Badu B Durham L III Joyce LD Joyce DL. Iatrogenic superior vena cava syndrome from percutaneous right ventricular assist device. *JTCVS Tech.* (2021) 6:92–4. 10.1016/j.xjtc.2020.11.013 34318155 PMC8300479

[289] Unger ED Sweis RN Bharat A. Unusual complication of a right ventricular support-extracorporeal membrane oxygenation cannula. *JAMA Cardiol.* (2021) 6:723–4. 10.1001/jamacardio.2021.0284 33729424 PMC8574132

[290] Anderson M Morris DL Tang D Batsides G Kirtane A Hanson I Outcomes of patients with right ventricular failure requiring short-term hemodynamic support with the Impella RP device. *J Heart Lung Transplant.* (2018) 37:1448–58. 10.1016/j.healun.2018.08.001 30241890

[291] Argaw ST Devlin PJ Clark JA Garza-Castillon R Kurihara C Bharat A. Fracture of dual lumen cannula leading to cerebrovascular accident in a patient supported with ECMO. *J Artif Organs.* (2022) 25:279–82. 10.1007/s10047-021-01306-z 35039962 PMC8763437

[292] Welp H Sindermann JR Deschka H Martens S Scherer M. Pulmonary bleeding during right ventricular support after left ventricular assist device implantation. *J Cardiothorac Vasc Anesth.* (2016) 30:627–31. 10.1053/j.jvca.2015.07.012 26460277

[293] Dandel M Javier MFDM Javier Delmo EM Loebe M Hetzer R. Weaning from ventricular assist device support after recovery from left ventricular failure with or without secondary right ventricular failure. *Cardiovasc Diagn Ther.* (2021) 11:226–42. 10.21037/cdt-20-288 33708495 PMC7944223

[294] Kremer J El-Dor A Sommer W Tochtermann U Warnecke G Karck M Long-term paracorporeal pulsatile mechanical circulatory support in adolescent and adult patients. *Interact Cardiovasc Thorac Surg.* (2022) 35:ivac107. 10.1093/icvts/ivac107 35532167 PMC9419688

[295] Michel S Buchholz S Buech J Veit T Fabry T Abicht J Bridging patients in cardiogenic shock with a paracorporeal pulsatile biventricular assist device to heart transplantation-a single-centre experience. *Eur J Cardiothorac Surg.* (2022) 61:942–9. 10.1093/ejcts/ezab547 35020902

[296] Bartfay S-E Dellgren G Hallhagen S Wåhlander H Dahlberg P Redfors B Durable circulatory support with a paracorporeal device as an option for pediatric and adult heart failure patients. *J Thorac Cardiovasc Surg.* (2021) 161:1453–64.e4. 10.1016/j.jtcvs.2020.04.163 32653285

[297] Shah P Ha R Singh R Cotts W Adler E Kiernan M Multicenter experience with durable biventricular assist devices. *J Heart Lung Transplant.* (2018) 37:1093–101. 10.1016/j.healun.2018.05.001 30173824

[298] Farag J Woldendorp K McNamara N Bannon PG Marasco SF Loforte A Contemporary outcomes of continuous-flow biventricular assist devices. *Ann Cardiothorac Surg.* (2021) 10:311–28. 10.21037/acs-2021-cfmcs-34 34159113 PMC8185384

[299] Maynes EJ O’Malley TJ Luc JGY Weber MP Horan DP Choi JH Comparison of SynCardia total artificial heart and HeartWare HVAD biventricular support for management of biventricular heart failure: a systematic review and meta-analysis. *Ann Cardiothorac Surg.* (2020) 9:69–80. 10.21037/acs.2020.03.07 32309154 PMC7160621

[300] Krabatsch T Potapov E Stepanenko A Schweiger M Kukucka M Huebler M Biventricular circulatory support with two miniaturized implantable assist devices. *Circulation.* (2011) 124(11 Suppl.):S179–86. 10.1161/CIRCULATIONAHA.110.011502 21911810

[301] Tran HA Pollema TL Silva Enciso J Greenberg BH Barnard DD Adler ED Durable biventricular support using right atrial placement of the HeartWare HVAD. *ASAIO J.* (2018) 64:323–7. 10.1097/MAT.0000000000000645 28841580

[302] Marasco S Simon AR Tsui S Schramm R Eifert S Hagl CM International experience using a durable, centrifugal-flow ventricular assist device for biventricular support. *J Heart Lung Transplant.* (2020) 39:1372–9. 10.1016/j.healun.2020.08.006 32917479

[303] Shehab S Macdonald PS Keogh AM Kotlyar E Jabbour A Robson D Long-term biventricular HeartWare ventricular assist device support–case series of right atrial and right ventricular implantation outcomes. *J Heart Lung Transplant.* (2016) 35:466–73. 10.1016/j.healun.2015.12.001 26849954

[304] Daneshmand MA Bishawi M Milano CA Schroder JN. The HeartMate 6. *ASAIO J.* (2020) 66:e46–9. 10.1097/MAT.0000000000001011 31045916 PMC6925356

[305] Mohan S David P Gu K Noah M Ms S Anelechi A HeartMate 6: bridge to heart transplantation utilizing biventricular support for cardiogenic shock. *J Am Coll Cardiol.* (2022) 79(9 Suppl.):2701. 10.1016/S0735-1097(22)03692-0

[306] Hanke JS Dogan G Haverich A Schmitto JD. Implantation of two HeartMate 3s in the setting of a total artificial heart. *Operat Tech Thorac Cardiovasc Surg.* (2021) 26:67–80. 10.1053/j.optechstcvs.2020.10.002

[307] McGiffin D Kure C McLean J Marasco S Bergin P Hare JL The results of a single-center experience with HeartMate 3 in a biventricular configuration. *J Heart Lung Transplant.* (2021) 40:193–200. 10.1016/j.healun.2020.12.006 33423854

[308] Lavee J Mulzer J Krabatsch T Marasco S McGiffin D Garbade J An international multicenter experience of biventricular support with HeartMate 3 ventricular assist systems. *J Heart Lung Transplant.* (2018) 37:1399–402. 10.1016/j.healun.2018.08.008 30241889

[309] Rame JE Pagani FD Kiernan MS Oliveira GH Birati EY Atluri P Evolution of late right heart failure with left ventricular assist devices and association with outcomes. *J Am Coll Cardiol.* (2021) 78:2294–308. 10.1016/j.jacc.2021.09.1362 34857091

[310] McNamara N Narroway H Williams M Brookes J Farag J Cistulli D Contemporary outcomes of continuous-flow left ventricular assist devices-a systematic review. *Ann Cardiothorac Surg.* (2021) 10:186–208. 10.21037/acs-2021-cfmcs-35 33842214 PMC8033255

[311] King PM Raymer DS Shuster J Crain M Bhatia A Hartupee J Right heart failure while on left ventricular assist device support is associated with primary graft dysfunction. *ASAIO J.* (2020) 66:1137–41. 10.1097/MAT.0000000000001156 33136601

[312] Ruiz-Cano MJ Ramazyan L Schramm R Lauenroth V Paluszkiewicz L Rojas S Clinical implications of late-onset right ventricular failure after implantation of a continuous-flow left ventricular assist device as bridge to transplantation. *Eur J Cardiothorac Surg.* (2021) 60:177–85. 10.1093/ejcts/ezab114 33783490

[313] Rich JD Gosev I Patel CB Joseph S Katz JN Eckman PM The incidence, risk factors, and outcomes associated with late right-sided heart failure in patients supported with an axial-flow left ventricular assist device. *J Heart Lung Transplant.* (2017) 36:50–8. 10.1016/j.healun.2016.08.010 27746085

[314] Brener MI Hamid NB Fried JA Masoumi A Raikhelkar J Kanwar MK Right ventricular pressure-volume analysis during left ventricular assist device speed optimization studies: insights into interventricular interactions and right ventricular failure. *J Card Fail.* (2021) 27:991–1001. 10.1016/j.cardfail.2021.04.019 33989781

[315] Sack KL Dabiri Y Franz T Solomon SD Burkhoff D Guccione JM. Investigating the role of interventricular interdependence in development of right heart dysfunction during LVAD support: a patient-specific methods-based approach. *Front Physiol.* (2018) 9:520. 10.3389/fphys.2018.00520 29867563 PMC5962934

[316] Shah P Badoe N Phillips S Abdullah K May CW Nabut JL Unrecognized left heart failure in LVAD recipients: the role of routine invasive hemodynamic testing. *ASAIO J.* (2018) 64:183–90. 10.1097/MAT.0000000000000617 28665826

[317] Uriel N Burkhoff D Rich JD Drakos SG Teuteberg JJ Imamura T Impact of hemodynamic ramp test-guided HVAD speed and medication adjustments on clinical outcomes. *Circ Heart Fail.* (2019) 12:e006067. 10.1161/CIRCHEARTFAILURE.119.006067 30946600

[318] Imamura T Nguyen A Kim G Raikhelkar J Sarswat N Kalantari S Optimal haemodynamics during left ventricular assist device support are associated with reduced haemocompatibility-related adverse events. *Eur J Heart Fail.* (2019) 21:655–62. 10.1002/ejhf.1372 30592363 PMC7147872

[319] Uriel N Morrison KA Garan AR Kato TS Yuzefpolskaya M Latif F Development of a novel echocardiography ramp test for speed optimization and diagnosis of device thrombosis in continuous-flow left ventricular assist devices: the Columbia ramp study. *J Am Coll Cardiol.* (2012) 60:1764–75. 10.1016/j.jacc.2012.07.052 23040584 PMC3545519

[320] Kanemaru E Yoshitani K Fukushima S Fujita T Ohnishi Y. Effect of left ventricular assist device implantation on right ventricular function: assessment based on right ventricular pressure-volume curves. *Artif Organs.* (2020) 44:1192–201. 10.1111/aor.13749 32530056

[321] Couperus LE Delgado V Khidir MJH Vester MPM Palmen M Fiocco M Pump speed optimization in stable patients with a left ventricular assist device. *ASAIO J.* (2017) 63:266–72. 10.1097/MAT.0000000000000483 27922889

[322] William J Mak V Leet A Kaye DM Nanayakkara S. Optimal mechanical unloading in left ventricular assist device recipients relates to progressive up-titration in pump speed. *J Am Soc Echocardiogr.* (2020) 33:583–93. 10.1016/j.echo.2020.01.002 32173204

[323] Grupper A Kodesh A Lavee J Fefer P Barbash IM Elian D Diastolic plateau – Invasive hemodynamic marker of adverse outcome among left ventricular assist device patients. *Front Cardiovasc Med.* (2022) 9:847205. 10.3389/fcvm.2022.847205 35433856 PMC9008249

[324] Imamura T Nitta D Fujino T Smith B Kalantari S Nguyen A Deep Y-descent in right atrial waveforms following left ventricular assist device implantation. *J Card Fail.* (2020) 26:360–7. 10.1016/j.cardfail.2020.01.004 31935459 PMC7141956

[325] Frea S Centofanti P Pidello S Giordana F Bovolo V Baronetto A Noninvasive assessment of hemodynamic status in HeartWare left ventricular assist device patients: validation of an echocardiographic approach. *JACC Cardiovasc Imaging.* (2019) 12(7 Pt 1):1121–31. 10.1016/j.jcmg.2018.01.026 29550313

[326] Kanwar MK Selzman CH Ton V-K Miera O CornwellIII WK Antaki J Clinical myocardial recovery in advanced heart failure with long term left ventricular assist device support. *J Heart Lung Transplant.* (2022) 41:1324–34. 10.1016/j.healun.2022.05.015 35835680 PMC10257189

[327] Fujino T Imamura T Nguyen A Chung B Raikhelkar J Rodgers D Short-term efficacy and safety of tolvaptan in patients with left ventricular assist devices. *ASAIO J.* (2020) 66:253–7. 10.1097/MAT.0000000000001079 31567410 PMC7340106

[328] Konstam MA Kiernan MS Bernstein D Bozkurt B Jacob M Kapur NK Evaluation and management of right-sided heart failure: a scientific statement from the American Heart Association. *Circulation.* (2018) 137:e578–622. 10.1161/CIR.0000000000000560 29650544

[329] Mullens W Damman K Harjola V-P Mebazaa A Brunner-La Rocca H-P Martens P The use of diuretics in heart failure with congestion – A position statement from the Heart Failure Association of the European Society of Cardiology. *Eur J Heart Fail.* (2019) 21:137–55. 10.1002/ejhf.1369 30600580

[330] Anyanwu EC Kagan V Bhatia A Tehrani DM Adatya S Kim G Home inotropes in patients supported with left ventricular assist devices. *ASAIO J.* (2019) 65:e7–11. 10.1097/MAT.0000000000000753 29461278

[331] Yalcin YC Caliskan K. Intermittent levosimendan treatment for late onset right ventricular failure in a patient supported with a left ventricular assist device. *Artif Organs.* (2020) 44:533–4. 10.1111/aor.13608 31838742

[332] Uriel N Burkhoff D Kim G Silverstein T Juricek C Kaye DM Oral milrinone for the treatment of chronic severe right ventricular failure in left ventricular assist device patients. *Circ Heart Fail.* (2021) 14:e007286. 10.1161/CIRCHEARTFAILURE.120.007286 33736460

